# Induction of Metastatic Gastric Cancer by Peroxisome Proliferator-Activated Receptor*δ* Activation

**DOI:** 10.1155/2010/571783

**Published:** 2010-12-27

**Authors:** Claire B. Pollock, Olga Rodriguez, Philip L. Martin, Chris Albanese, Xin Li, Levy Kopelovich, Robert I. Glazer

**Affiliations:** ^1^Department of Oncology, Lombardi Comprehensive Cancer Center, Washington, DC 20057, USA; ^2^Center for Advanced Preclinical Research, SAIC/NCI-Frederick, Frederick, MD 21702, USA; ^3^Department of Biostatistics, Bioinformatics, and Biomathematics, Lombardi Comprehensive Cancer Center, Washington, DC 20057, USA; ^4^Chemoprevention Agent Development and Research Group, Division of Cancer Prevention, National Cancer Institute, Bethesda, MD 20814, USA

## Abstract

Peroxisome proliferator-activated receptor*δ* (PPAR*δ*) regulates a multiplicity of physiological processes associated with glucose and lipid metabolism, inflammation, and proliferation. One or more of these processes likely create risk factors associated with the ability of PPAR*δ* agonists to promote tumorigenesis in some organs. In the present study, we describe a new gastric tumor mouse model that is dependent on the potent and highly selective PPAR*δ* agonist GW501516 following carcinogen administration. The progression of gastric tumorigenesis was rapid as determined by magnetic resonance imaging and resulted in highly metastatic squamous cell carcinomas of the forestomach within two months. Tumorigenesis was associated with gene expression signatures indicative of cell adhesion, invasion, inflammation, and metabolism. Increased PPAR*δ* expression in tumors correlated with increased PDK1, Akt, *β*-catenin, and S100A9 expression. The rapid development of metastatic gastric tumors in this model will be useful for evaluating preventive and therapeutic interventions in this disease.

## 1. Introduction

Gastric cancer is the second leading cause of cancer-related death globally, particularly in developing countries, due to its metastatic nature at the time of diagnosis [1, 2]. Among the many risk factors for gastric cancer are diet, smoking, and inflammation associated with *H. pylori* infection [3–5] that are likely exacerbated in patients with proinflammatory gene polymorphisms [6]. 

PPARs are ligand-dependent nuclear receptors that regulate expression of a multiplicity of genes associated with metabolic disorders, such as type II diabetes and lipodystrophies [7, 8]. PPARs consist of the *α*, *γ* and *δ* isotypes that regulate not only glucose and lipid metabolism, but also proliferation, inflammation, and angiogenesis [9–13]. PPAR*δ* expression is increased in breast, colon, and head and neck cancers [9, 14–17] and is associated with a more aggressive phenotype in breast cancer cells [18]. The selective PPAR*δ* agonist GW501516 acts as a tumor promoter in mammary carcinogenesis [19] and colon tumorigenesis [15, 20, 21], whereas disruption of PPAR*δ* expression blocks mammary [22] and colon tumorigenesis [23, 24]. PPAR*δ* is regulated by risk factors implicated in cancer progression, including K-Ras [25], Wnt [26], and Pges/Cox2 [22, 27], and is associated with activation of angiogenesis [20, 28–30]. PPAR*δ* regulates proinflammatory signaling through NF*κ*B and IL-1*β* [31], and is activated by metabolites of arachidonic acid metabolism that serve as PPAR ligands [14, 29, 32]. Notwithstanding these results, there are several reports showing that azoxymethane-induced colon carcinogenesis is increased in PPAR*δ* nullizygous mice [33, 34] and by the selective PPAR*δ* ligand GW0742 [35] (reviewed in [36]). Differences between the various nullizygous models that may account for some of these disparities have been discussed [9, 21].

Here we demonstrate that activation of PPAR*δ* by a selective agonist, GW501516, rapidly induces highly metastatic gastric tumors following carcinogen administration, which expressed a markedly increased inflammatory gene expression signature. These findings suggest a crucial role for PPAR*δ* in the progression of this disease.

## 2. Materials and Methods

### 2.1. Materials

GW501516 was synthesized as previously described [37] and was provided by the Chemoprevention Branch, National Cancer Institute, NIH, Bethesda, MD.

### 2.2. Carcinogenesis

DMBA (Sigma-Aldrich, St Louis, MO) was dissolved in cottonseed oil at a concentration of 10 mg/ml, and six week-old female FVB mice were administered 4 weekly doses of 1 mg DMBA by gavage. Animals were fed a standard diet or a diet supplemented with 0.005% (w/w) GW501516 beginning one day after the last dose of DMBA as previously described [19]. Mice were euthanized when gastric tumors reached ≥2cm^3^ as determined by MRI imaging or if mice became moribund. All protocols were approved by the Georgetown University Animal Care and Use Committee.

### 2.3. Magnetic Resonance Imaging

MRI was performed on a 7.0 Tesla Bruker horizontal spectrometer/imager with a 20 cm bore equipped with 100 gauss/cm microimaging gradients and run by Paravision 4.0 software in the Preclinical Imaging Research Laboratory, Lombardi Comprehensive Cancer Center. Mice were anesthetized using 1.5% isoflurane and 30% nitrous oxide, positioned in a custom-made stereotaxic animal holder with temperature and respiration control, and imaged in a 72 mm birdcage radiofrequency volume coil as previously described [38]. The imaging protocol used was a T2-weighted RARE (rapid acquisition with refocused echos) two-dimensional sequence with the following parameters: Matrix: 256, TR: 5822 msec, TE: 36 msec, number of averages: 4, RARE factor: 8, FOV: 4.0 cm, number of slices: 4, slice thickness: 0.5 mm, and with respiratory gating to account for breathing movement.

### 2.4. Histopathology and Immunohistochemistry (IHC)

Stomach and tumors were excised, and formalin-fixed, paraffin-embedded sections were prepared for H&E staining and IHC. Antigen retrieval was carried out by incubation of tissue sections in 10 mM sodium citrate buffer (pH 6.0) for 20 min at a subboiling temperature in an electric steamer as previously described [19, 39]. Endogenous peroxidase activity was quenched with 3% hydrogen peroxide for 10 min and incubated for 30 min with blocking solution (10% goat serum in Tris-buffered saline), followed by incubation overnight at 4°C with the appropriate primary antibody diluted in blocking solution. Biotin-conjugated secondary antibodies were diluted in TBS containing 0.1% Tween-20 and incubated for 30 min at room temperature using the ABC Vectastain (Vector Laboratories) detection system and diaminobenzidine (Pierce), and slides were counterstained with Harris-modified hematoxylin (Fisher Scientific) and dehydrated and mounted in Permount (Fisher Scientific). The following antibodies and their dilutions were used: anti-CK14 (1 : 200, ms-115-P1, Neomarkers), anti-CK18 (1 : 200, sc-7197, Santa Cruz Biotechnology), PDK1 (1 : 50, sc-17765, Santa Cruz Biotechnology), anti-pS473 Akt (1 : 200, 40515, Cell Signaling Biotechnology), anti-*β*-catenin (1 : 50, sc-7963, Santa-Cruz Biotechnology) and anti-S100a9 (1 : 50, sc-65580, Santa Cruz Biotechnology).

### 2.5. Gene Microarray Analysis

Three groups of tissues were analyzed: (1) gastric tumors, (2) forestomach (nonglandular) from GW501516-treated mice, and (3) forestomach from DMBA-treated mice. Tissue was excised, washed in phosphate-buffered saline, and stored in RNAlater (Ambion) at *‒*20°C until RNA extraction. Tissue was snap-frozen in liquid nitrogen, pulverized in a mortar and pestle, and RNA extracted using an RNeasy Mini Kit (Qiagen) according to the manufacturer's protocol. RNA purity was assessed by an A_260_/A_280_ ratio of ≥1.9, and by the integrity of 18S and 28S rRNA using an Agilent microfluidic chip. Array analysis was carried out on cRNA prepared from equal amounts of RNA (1 *μ*g) pooled from 5 mice per group as previously described [19, 40]. Biotin-labeled cRNA was fragmented at 94°C for 35 min and hybridized overnight to an Affymetrix mouse 430A 2.0 GeneChip representing approximately 14,000 annotated mouse genes. GeneChips were scanned with an Agilent Gene Array scanner, and grid alignment and raw data generation with the Affymetrix GeneChip Operating software 1.1. A noise value (*Q*) based on the variance of low-intensity probe cells was used to calculate a minimum threshold for each GeneChip. Samples were averaged and data refined by eliminating genes with signal intensities <300 in both comparison groups, and heat maps were generated from ≥3-fold changes in gene expression normalized to untreated forestomach using unsupervised hierarchical cluster analysis as previously described [41]. Gene ontology analysis utilized Ariadne Pathway Studio version 7.1

### 2.6. Quantitative Real-Time Polymerase Chain Reaction (qRT-PCR)

Total RNA was extracted as described above, and equal amounts of RNA (1 *μ*g) were pooled from each group (five samples per group) and reverse transcribed with the Omniscript RT kit (Qiagen) in a total volume of 20 *μ*L as previously described [19, 39]. PCR was performed in triplicate using an ABI-Prism 7700 (Applied Biosystems, Foster City, CA) with SYBRGreen I detection (Qiagen) according to the manufacturer's protocol. Amplification using the appropriate primers (see Table S1 in supplementary material available at doi:10.1155/2010/571783) was confirmed by ethidium bromide staining of the PCR products on an agarose gel. The expression of each target gene was normalized to GAPDH and is presented as the ratio of the target gene to GADPH expression calculated using the formula, 2^−ΔCt^, where ΔCt = Ct^Target^ − Ct^18s^.

## 3. Results

### 3.1. PPAR*δ* Agonist GW501516 Rapidly Promotes Gastric Tumorigenesis

**Figure 1:**
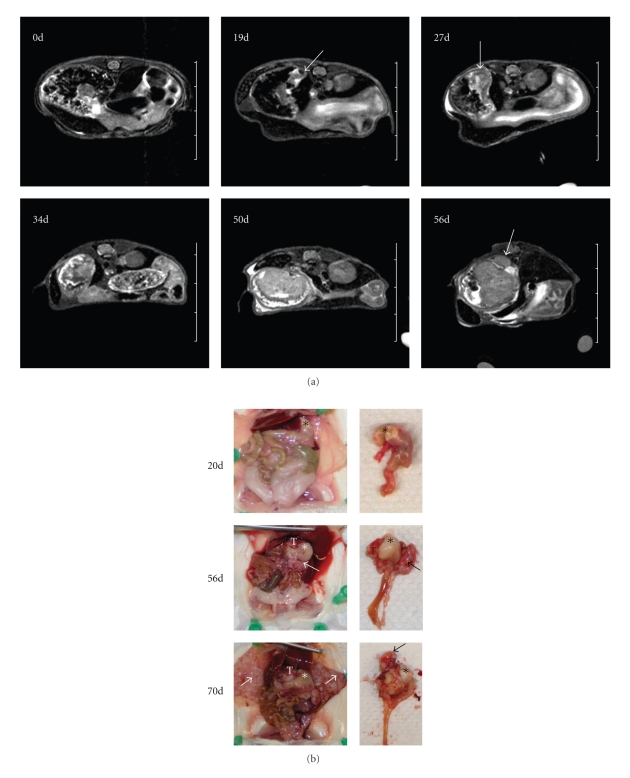
PPAR*δ* agonist GW501516 induces gastric tumorigenesis after DMBA treatment. (a) *In vivo* axial T2-weighted abdominal MR Images showing gastric tumor progression at the indicated time points; *d*, number of days animals were administered the GW501516-supplemented diet. Tumor growth initiated in the forestomach (*arrow) *at 19 days, rapid tumor growth between 27 and 50 days, and tumor invasion through the stomach (*arrow*) at 56 days. (b) Gross morphology of tumors at indicated time points. Forestomach (*), gastric tumors (*T*) and metastases (*arrows*) are indicated. Tumor is confined within the stomach at 20 days. Invasion of primary tumor through the stomach (*T*) and metastases (*arrow*) were evident at 56 days, and intraperitoneal metastases along the abdominal wall (*white arrows*) occurred at 70 days.

Mice maintained on a diet supplemented with PPAR*δ* agonist GW501516 following carcinogen administration resulted in the rapid development of gastric tumors in 12/15 animals, whereas treatment with either GW501516 or DMBA alone was not tumorigenic (Table 1). To follow the onset and progression of tumorigenesis more precisely, five mice were monitored by MRI (Figure 1(a)). Tumors were visible as early as 19 days after beginning the GW501516 diet and appeared to initiate in the forestomach (Figure 1(a)). By 50 days, tumor had filled the stomach lumen, and by 56 days it had extravasated through the gastric wall (Figure 1(a)). Gross inspection of the stomach confirmed that the tumor was confined within the stomach at day 20 but had invaded through the stomach wall forming local metastases by day 56 (Figure 1(b)). Mice showed signs of morbidity between days 63 and 70 (mean survival 67 days), where metastases were present throughout the mesentery and adjacent serosal organ surfaces including the abdominal wall (Figure 1(b)).

**Figure 2:**
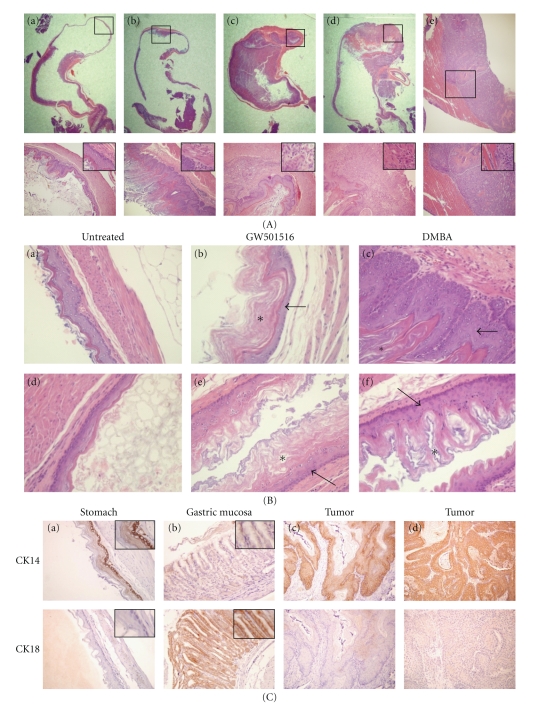
Pathophysiology of gastric tumorigenesis. (A) Histological changes in the forestomach after GW501516 and/or DMBA treatment. *Upper panel:* Stereoscopic images of H&E stained sections of the stomach in (a) untreated, (b) DMBA-treated, (c, d) DMBA plus GW501516-treated mice, and (e) metastases of the abdominal wall. *Lower panel*: higher magnification (200x) of the boxes in areas in the *upper panel*. *Insets*, magnification 400x. (a) Morphology of the normal forestomach wall. (b) Squamous epithelial hyperplasia, where the basal membrane is well defined. (c) Squamous cell carcinoma. (d) Invasive squamous cell carcinoma showing disruption of the basement membrane. (e) Metastatic squamous carcinoma showing invasion into the abdominal wall. (B) Histological changes in the stomach and esophagus after treatment with either GW501516 or DMBA. H&E sections of the forestomach wall (a, b, c) and esophagus (d, e, f) six months after administration of GW501516 or five months after treatment with DMBA. Both GW501516 and DMBA were associated with increased keratinization (*) of squamous epithelium (arrow). Magnification 400x. (C) Cytokeratin 14 (*CK14*) and cytokeratin18 (*CK18*) expression in gastric tissue and tumors. Squamous epithelium of the forestomach (a), gastric mucosa (b), and gastric tumors (c, d). Tumors are CK14^+^/CK18^−^ indicating a squamous epithelial origin. Magnification 200x, *insets*, 400x.

Primary tumors and metastases were uniformly squamous cell carcinomas with varying degrees of keratinization (Figure 2(A)). Animals fed the GW501516 diet for six months without prior DMBA treatment did not exhibit hyperplasia or dysplasia (Figure 2(B)), and DMBA treatment alone produced squamous cell hyperplasia of the forestomach without signs of dysplasia (Figures 2(A) and 2(B)). No changes occurred in the gastric mucosa resulting from DMBA and GW501516 treatment alone (data not shown), and esophageal squamous epithelium was unaffected by DMBA treatment (Figure 2(B)). Gastric tumors were positive for the squamous basal cell marker CK14, and negative for the columnar epithelial cell marker CK18 [42] (Figure 2(C)).

### 3.2. Differential Gene Expression in Gastric Tumors and Stomach

**Figure 3:**
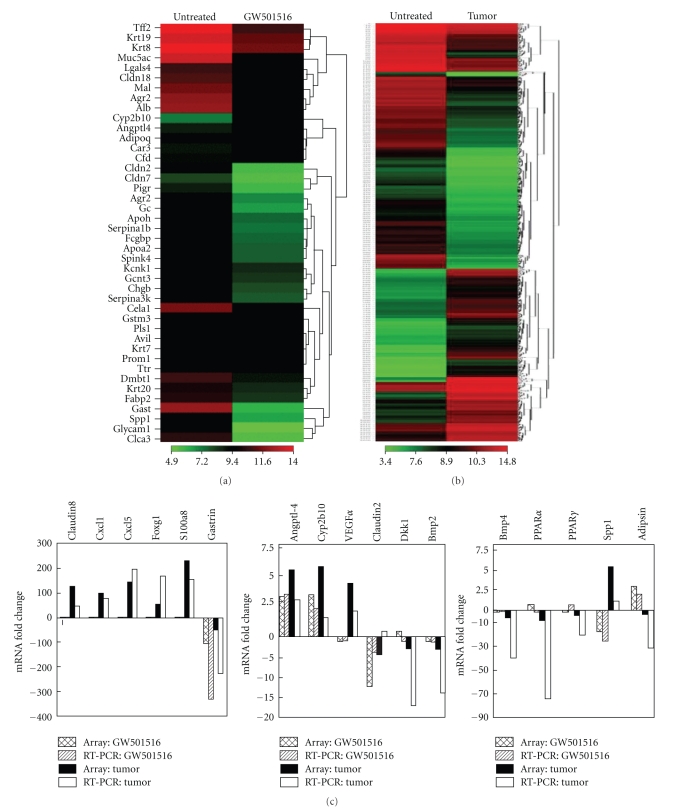
Differential gene expression in GW501516-treated stomach and gastric tumors. Gene microarray analysis was carried out with pooled RNA samples prepared from either five tissue samples of forestomach from untreated and GW501516 treated mice or six gastric tumors. Heat maps represent unsupervised hierarchical clustering of ≥3-fold changes in signal intensity normalized to untreated forestomach. (a) Heatmap of GW501516-treated versus stomach representing 42 genes. (b) Tumor versus stomach representing 811 genes. (c) qRT-PCR analysis of the relative changes in gene expression in the stomach after GW501516 treatment (*GW501516*), and in tumors (*Tumor*). GW501516 treated: array,* cross-hatch*; qRT-PCR, *diagonal;* Tumors: array, *black*; qRT-PCR, *white*.

Tumors manifested a marked inflammatory phenotype denoted by increased expression of chemokines Cxcl-1,-2,-5,-9,-14 and Ccl-2,-3,-8, S100a8, S100a9, and S100a3 and interleukins IL-1*β*, IL-6, and IL-24 (Table 2, Figure 3(b), and Table S2). In addition to these changes, increased expression of prostaglandin metabolism genes Ptgs2/Cox2 and Ptges and reduced expression of PPAR*γ* and PPAR*α* were noted. To determine if these changes were tumor specific, gene expression was assessed in stomach tissue after treatment with either GW501516 for seven days (Figure 3(a), Table S3) or DMBA for four weeks (Table S4). GW501516 increased expression of only five genes ≥3-fold, Angptl4, Cyp2b10, Cfd/Adipsin, Adipoq and Chi3l4 and markedly reduced expression of Gast, Ccla3, Glycam1, Spp1, Serpina1a, Cela1, Cldn2, and Fabp2 (Table S3). DMBA increased expression of S100a8, S100a9, and Ccl8 4–10-fold and reduced expression of the same subset of genes as GW501516 (Table S4). Thus, changes in Gast, Ccla3, Glycam1, Spp1, Serpina1a, Cela1, Cldn2, and Fabp2 are a result of GW501516 treatment and are not tumor specific. On the other hand, DMBA produced an inflammatory response denoted by increase S100A8, Ccl8, and S100A9 although it was an order of magnitude less than in tumors. The increase in Krt6a by DMBA is consistent with increased keratinization in the stomach (Figure 2(B)) but was less pronounced than in tumors. Real-time qRT-PCR analysis generally confirmed the changes in expression of several genes, including Cldn8, Cxcl1, Cxcl5, Foxg1, S100a8, Angptl4, Cyp2b10, Vegf*α* and Spp1, Gast, Dkk1, Bmp3, Bmp4, PPAR*α*, and PPAR*γ* (Figure 3(c)). 

**Figure 4:**
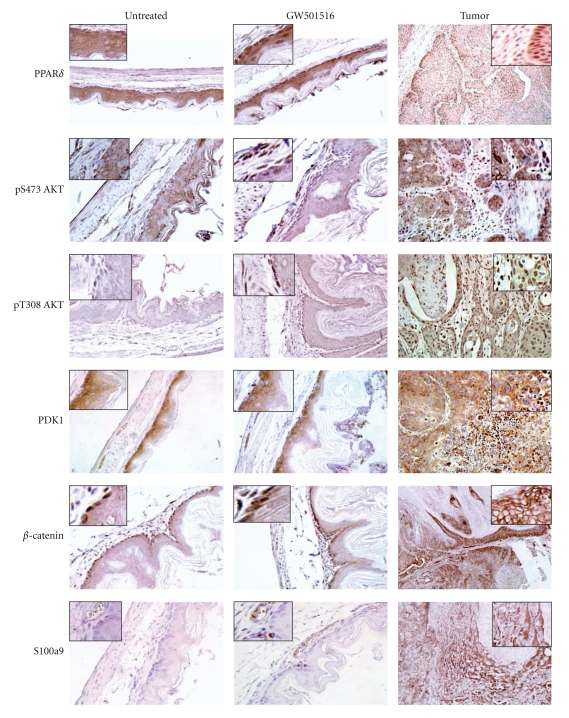
Immunohistochemical analysis of forestomach after GW501516 treatment and gastric tumors. IHC detection of PPAR*δ*, pS473Akt, pT308 Akt, PDK1, *β*-catenin, and S100a9. Magnification: untreated and GW501516 treated, 400x; tumor, PPAR*δ*, PDK1, and *β*-catenin, 200x; pT308Akt, pS473Akt, S100a9, and 400x. *Inset*, 2x original magnification. PPAR*δ* shows diffuse reactivity in untreated gastric squamous epithelium, increased nuclear localization after GW501516 treatment, and strong nuclear expression in tumor and stromal cells (inset). pS473Akt and pT308Akt expressed weak diffuse reactivity in untreated gastric tissue, increased staining in basal cells and the submucosal cell layer after GW501516 treatment, and strong reactivity in tumor and stromal cells. PDK1 exhibited diffuse cytoplasmic localization throughout the untreated squamous epithelium and was unchanged after GW501516 treatment, whereas PDK1 was increased in tumors similarly to pS473Akt and pT308Akt. Nuclear *β*-catenin was present in basal cells of untreated squamous epithelium and was unchanged after GW501516 treatment, whereas tumors expressed increased *β*-catenin at cellular junctions (inset). S100a9 was undetectable in untreated tissue and was increased in blood vessels and squamous epithelium (inset), whereas tumors exhibited increased diffuse cytoplasmic staining.

 The expression of PPAR*δ* and factors known to be associated with its signaling were assessed in tumors and forestomach after GW501516 treatment (Figure 4). GW501516 increased nuclear localization of PPAR*δ* in gastric squamous epithelium and tumors, in contrast to its diffuse cytoplasmic staining in untreated gastric tissue. GW501516 also elicited strong pS473Akt and pT308Akt staining in basal cells and in the submucosal layer, as well as in tumor and stromal tissue, which correlated with more intense PDK1 expression. *β*-Catenin was expressed in the nuclei of basal squamous epithelial cells and was not altered by GW501516 treatment, whereas tumors expressed increased *β*-catenin at cellular junctions. S100a9 was absent in untreated gastric epithelium but was expressed in endothelial and epithelial cells from GW501516-treated mice. Tumors expressed S100a9 in a diffuse pattern, with strong expression in blood vessels and adjacent epithelial cells.

## 4. Discussion

The present study describes a new model of metastatic gastric cancer that is dependent on the tumor promoting activity of PPAR*δ* agonist GW510516 following carcinogen administration. In contrast to a previous study reporting a low percentage of squamous cell carcinomas of the forestomach by DMBA [43], our DMBA regimen produced only forestomach hyperplasia without signs of dysplasia up to five months after treatment (Figure 2(B)). This suggests a high sensitivity of mouse forestomach squamous epithelium to dysplasia, and the predilection of GW501516 to promote tumors of this histotype [19]. This model differs from N-methyl-N-nitrosourea-induced gastric tumors in wild-type and APC^Min^ transgenic mice [44, 45] in that it is dependent on both DMBA-induced mutagenesis and the tumor-promoting effects of GW501516. A feature of this model is its short latency of approximately three weeks in comparison to 10 to 20 weeks for NMU-treated wild-type and APC^Min^ mice. An important histopathological distinction, and perhaps disadvantage of the GW501516 tumor model, is that it produces squamous cell carcinomas from the nonglandular forestomach rather than adenocarcinomas from the glandular tissue that comprises the majority of human gastric cancer [46]. Since this model was dependent on the selective PPAR*δ* agonist GW501516 [47], it is important to note that the dose of GW501516 used in the present and previous studies [19] is equivalent to daily oral doses of 3–10 mg/kg that were previously shown to specifically enhance PPAR*δ*-dependent fatty acid oxidation in mice [48]. In addition, PPAR*δ* agonist GW7042, which is almost identical to GW501516 in structure, potency, and specificity, was inactive in inducing gene expression in PPAR*δ* knockout mice [49], suggesting that the tumor promoting effects of GW501516 and GW7042 are not due to off-target effects. 

**Figure 5:**
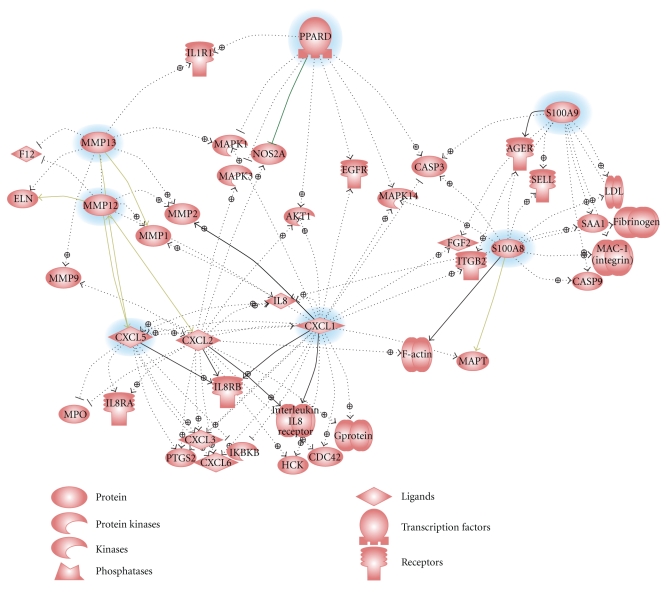
Signaling networks associated with PPAR*δ* and inflammatory and invasive gene expression. Common signaling pathways were analyzed for six genes with the greatest changes in expression in gastric tumors (highlighted in *blue*) versus forestomach using Ariadne Pathway Studio 7.1.

Tumors induced by GW501516 exhibited a distinct inflammatory gene expression signature comprised predominantly of chemokine, MMP, and S100 genes (Table S2). This was unexpected in view of the lack of a similar signature after treatment with GW501516 (Table S3), and the fact that GW501516 induces an anti-inflammatory response in macrophages [50] and protects the heart against oxidative stress [51, 52]. Gene ontology analysis of gene expression in the gastric tumors indicates that PPAR*δ*, MMP12, MMP13, Cxcl1, Cxcl5, S100A8, and S100A9 share both common and disparate pathway interactions that likely contributed to the tumorigenic phenotype (Figure 5). PPAR*δ* is associated with activation of genes related to proliferation (EGFR, Akt1) and adhesion (Itgb2), whereas S100A9 is associated with angiogenesis (Fgf2) and inflammation (Ager). Cxcl1 activates proliferation (Mapk3, Mapk14, and Akt1), angiogenesis (Fgf2), and invasion (MMP2, MMP9), and Cxcl5 activates other chemokines (Cxcl1, Cxcl2, and Cxcl3) and Ptgs2/Cox2. This scheme reiterates the ability of S100A8 and S100A9 to act as ligands for Ager (advanced glycation end-product receptor), which mediates acute and chronic inflammation, tumor development, and metastasis [53, 54]. This paradigm is also consistent with GW501516-induced activation of Ptges and Ptgs2/Cox-2 expression [55, 56], which initiate the production of prostacyclins [14] and arachidonic acid metabolites [29] that serve as PPAR*δ* ligands [57]. Since Cox-2 inhibitors reduce inflammation-related gastrointestinal carcinogenesis [58], and overexpression or deletion of Ptgs2 increases or suppresses tumorigenesis, respectively [59, 60]; this suggests cooperativity between PPAR*δ* and inflammatory signaling pathways in gastric tumorigenesis. The ability of PPAR*δ* to have an anti-inflammatory effect in normal cells [51, 52] and a proinflammatory effect in tumors is reminiscent of the dual roles of TGF-*β* in tumor cells [61, 62]. TGF*β* can function as a proinflammatory cytokine to activate S100A8 and S100A9 expression in the presence of activated Ras [63] but acts as a repressor of inflammation-induced PPAR*δ* expression in normal cells [64, 65]. The increased expression of the PPAR*δ* target gene, Agptl4, the TGF*β*-activated genes Runx1 and Runx2, and S100A8 and S100A9 in the gastric tumors indeed suggests a duality of function of both PPAR*δ* and TGF*β* signaling in gastric tumorigenesis.

PPAR*δ* is ubiquitously expressed in gastrointestinal tissue and gastric tumors [27], and GW501516 elicited increased PPAR*δ* nuclear staining and elevated pAkt in gastric epithelium and tumors. PPAR*δ*-dependant activation of Akt is required for the growth-promoting and antiapoptotic effects of PPAR*δ* [66–68], as shown by the delayed wound-healing response of PPAR*δ*-deficient keratinocytes [57, 69]. Enhanced Krt6a and Krt16 expression in tumors further suggests that PPAR*δ* plays an important role in gastric squamous cell differentiation and tissue renewal. 

Tumors also exhibited reduced PPAR*γ* and PPAR*α* expression that may have resulted, in part, from the negative regulation of PPAR*γ* by PPAR*δ* [70]. PPAR*γ* suppresses the growth and invasion of human colon [71] and gastric [72, 73] and esophageal carcinoma cells [74], and both PPAR*γ* [75] and PPAR*α* have anti-inflammatory actions [76]. Thus, reduction of PPAR*α* and PPAR*γ* expression may be an additional mechanism for facilitating the proinflammatory and tumor-promoting effects of GW501516. 

In summary, we describe a rapidly developing metastatic gastric cancer model dependent on the tumor-promoting effects of GW501516 following carcinogen treatment, which suggests a proinflammatory switch in PPAR*δ* function. This animal model will therefore be useful to delineate the role of PPAR*δ* in tumor initiation and progression and as a possible target for early intervention.

## Supplementary Material

Supplementary material contains qRT-PCR (Table S1) primers and differential gene expression data in gastric tumors (Table S2), and in the stomach of GW501516-treated (Table S3) and DMBA-treated (Table S4) animals.

## Figures and Tables

**Table 1:** Gastric tumor occurrence after treatment with DMBA and GW501516.

Treatment group	No. animals	No. animals with tumors
DMBA	10	0
GW501516	10	0
DMBA + GW501516	15	12

**Table 2:** Differentially expressed genes in gastric tumors.

Gene Symbol	Gene Name	Fold Change
S100a8	S100 calcium-binding protein A8 (calgranulin A)	229.0
S100a9	S100 calcium-binding protein A9 (calgranulin B)	74.6
S100a3	chemokine (C-X-C motif) ligand 2	17.7
Cxcl2	chemokine (C-X-C motif) ligand 2	109.2
Cxcl5	chemokine (C-X-C motif) ligand 5	145.6
Cxcl1	chemokine (C-X-C motif) ligand 1	100.0
Cxcl9	chemokine (C-X-C motif) ligand 9	13.3
Ccl2	chemokine (C-C motif) ligand 2	12.8
Ccl3	chemokine (C-C motif) ligand 3	28.7
Ccl8	chemokine (C-C motif) ligand 8	13.4
Il1b	interleukin 1 beta	20.9
Il24	interleukin 24	23.7
Il6	interleukin 6	39.4
Mmp10	matrix metallopeptidase 10	15.8
Mmp12	matrix metallopeptidase 12	104.1
Mmp13	matrix metallopeptidase 13	62.5
Mmp3	matrix metallopeptidase 3	9.3
Mmp9	matrix metallopeptidase 9	6.0
Ppara	peroxisome proliferator activated receptor alpha	−8.6
Pparg	peroxisome proliferator activated receptor gamma	−4.3
Ptges	prostaglandin E synthase	11.4
Ptgs2	prostaglandin-endoperoxide synthase 2	14.1
Krt16	keratin 16	116.0
Krt6a	keratin 6A	86.2
