# Supplementary material for: Induction of Metastatic Gastric Cancer by Peroxisome Proliferator-Activated Receptorδ Activation
Source: PPAR Res. 2010 Dec 27;2010:571783. doi: 10.1155/2010/571783 (PMC3026990; doi:10.1155/2010/571783)
Supplement: Supplementary file 1 — Supplementary material contains qRT-PCR (Table S1) primers and differential gene expression data in gastric tumors (Table S2), and in the stomach of GW501516-treated (Table S3) and DMBA-treated (Table S4) animals. [file 571783.f1.pdf]

TABLE S1. qRT-PCR primers

Name	Accession #	Forward primer	Reverse primer
Adipsin (Cfd	NM_013459	5'-CAATCATGAACCGGACAA	5'-CGCGAGAGCCCCACGTAACCACA
Angptl-4	NM_020581	5'-GACTTTTCCAGATCCAGCC	5'-CTCCGAAGCCATCCTTGTAG
BMP2	NM_007553	5'-CGCAGCTTCCATCACGAA	5'-TGCAGATGTGAGAAACTCGTC
BMP4	NM_007554	5'-ATTGCAGCTTTCTAGAGGT	5'-GGGAGCCAATCTTGAACAAAC
Claudin-2	NM_016675	5'-GCTTGTGACCCCTTGGAC	5'-CTCCTTACAAGTATCTGTGGGTG
Claudin 8	NM_018778	5'-AGCTGGATACAATTTGGGA	5'-CCACTGAGGCATGATAGTCAC
Cxcl1	NM_008176	5'-AACCGAAGTCATAGCCAC	5'-CAGACGGTGCCATCAGAG
Cxcl5	NM_009141	5'-GTTCCATCTCGCCATTCAT	5'-TTAAGCAAACACAACGCAGC
Cyp2b10	NM_009999	5'-GCTTTTCTGCCCTTCTCAA	5'-CTTGGGAGTGAGGTCAATGTC
Dkk3	NM_015814	5'-GAGATGTTTCGAGAGGTG	5'-TTGTGATAGTTGGGAGGTAAGC
Foxg1	NM_008241	5'-CCTGCCCTGTGAGTCTTTA	5'-CGTTCACTTACAGTCTGGTCC
Gastrin	NM_010257	5'-AACAGCCAACTATTCCCCA	5'-CCAGCACTAAGACCAGCATG
PPARalpha	NM_011144	5'-CATTTCCCTGTTTGTGGCT	5'-ATCTGGATGGTTGCTCTGC
PPARgamma	NM_011146	5'-TGTTATGGGTGAAACTCTC	5'-AGAGCTGATTCCGAAGTTGG
S100a8	NM_013650	5'-AGTGTCCTCAGTTTGTGCA	5'-ACTCCTTGTGGCTGTCTTTG
Spp1	NM 009263	5'-CCCATCTCAGAAGCAGAA	5'-TCATCATCGTCATCATCGTCG
Vegfa	NM_0010252	5'-GGCAGCTTGAGTTAAACG	5'-TGGTGACATGGTTAATCGGTC
GAPDH	NM_008084	5'-GCTCATGACCACAGTCCA	5'-TTGGCAGCACCAGTGGATG
