# Supplementary material for: Induction of Metastatic Gastric Cancer by Peroxisome Proliferator-Activated Receptorδ Activation
Source: PPAR Res. 2010 Dec 27;2010:571783. doi: 10.1155/2010/571783 (PMC3026990; doi:10.1155/2010/571783)
Supplement: Supplementary file 2 [file 571783.f2.pdf]

TABLE S2. Differentially expressed genes in gastric tumors compared to control stomach.

Gene Symbol	Probe set	Gene Name	Stomach	Tumor	Fold Change
Aadac	1448813_at	arylacetamide deacetylase (esterase)	1200.4	251.5	-4.8
Abca3	1451731_at	ATP-binding cassette, sub-family A (ABC1), member 3	1268.0	406.8	-3.1
Abca8a	1427371_at	ATP-binding cassette, sub-family A (ABC1), member 8a	1136.2	100.1	-11.3
Abcc9	1420408_a_at	ATP-binding cassette, sub-family C (CFTR/MRP), member 9	508.2	142.8	-3.6
Abhd14b	1451326_at	abhydrolase domain containing 14b	1034.7	261.6	-4.0
Abhd3	1417946_at	abhydrolase domain containing 3	409.7	81.9	-5.0
Abi3bp	1427053_at	ABI gene family, member 3 (NESH) binding protein	992.3	102.7	-9.7
Acad9	1429581_at	acyl-Coenzyme A dehydrogenase family, member 9	1215.7	389.7	-3.1
Acer1	1450825_at	alkaline ceramidase 1	1206.1	241.4	-5.0
Acot9	1418073_at	acyl-CoA thioesterase 9	917.6	2876.9	3.1
Acox2	1420673_a_at	acyl-Coenzyme A oxidase 2, branched chain	1285.6	153.6	-8.4
Acsbg1	1422428_at	acyl-CoA synthetase bubblegum family member 1	977.7	3092.2	3.2
Acsl4	1433531_at	acyl-CoA synthetase long-chain family member 4	403.9	1567.1	3.9
Acss1	1416617_at	acyl-CoA synthetase short-chain family member 1	741.5	202.6	-3.7
Acta1	1427735_a_at	actin, alpha 1, skeletal muscle	155.1	542.6	3.5
Acta2	1416454_s_at	actin, alpha 2, smooth muscle, aorta	25859.0	6008.7	-4.3
Actg2	1422340_a_at	actin, gamma 2, smooth muscle, enteric	14936.8	1495.1	-10.0
Ada	1417976_at	adenosine deaminase	20142.4	3300.0	-6.1
Adam12	1421172_at	a disintegrin and metallopeptidase domain 12 (meltrin alpha)	35.5	404.4	11.4
Adam8	1416871_at	a disintegrin and metallopeptidase domain 8	224.5	1709.1	7.6
Adh1	1416225_at	alcohol dehydrogenase 1 (class I)	4985.2	887.9	-5.6
Adh7	1421058_at	alcohol dehydrogenase 7 (class IV), mu or sigma polypeptide	2734.5	538.9	-5.1
Adipoq	1422651_at	adiponectin, C1Q and collagen domain containing	337.6	34.7	-9.7
Adm	1416077_at	adrenomedullin	149.5	2091.2	14.0
Agr2	1419268_at	anterior gradient 2 (Xenopus laevis)	5897.7	1844.1	-3.2
Aif1l	1424263_at	allograft inflammatory factor 1-like	1759.1	280.4	-6.3
Ak3	1432436_a_at	adenylate kinase 3	746.1	180.5	-4.1
Akap12	1419706_a_at	A kinase (PRKA) anchor protein (gravin) 12	1503.6	335.5	-4.5
Akr1c12	1422000_at	aldo-keto reductase family 1, member C12	1185.1	379.7	-3.1
Akr1c14	1418979_at	aldo-keto reductase family 1, member C14	673.1	160.1	-4.2
Akr1c18	1419136_at	aldo-keto reductase family 1, member C18	44.3	420.1	9.5
Akr1d1	1425771_at	aldo-keto reductase family 1, member D1	84.6	316.2	3.7
Alb	1425260_at	albumin	6338.0	239.1	-26.5
Alcam	1426300_at	activated leukocyte cell adhesion molecule	326.8	2177.7	6.7
Aldh1a1	1416468_at	aldehyde dehydrogenase family 1, subfamily A1	6978.9	1227.6	-5.7
Aldh1a3	1448789_at	aldehyde dehydrogenase family 1, subfamily A3	64.4	2802.6	43.5
Aldh1a7	1418601_at	aldehyde dehydrogenase family 1, subfamily A7	6880.1	1290.1	-5.3
Aldh3a1	1418752_at	aldehyde dehydrogenase family 3, subfamily A1	7508.8	643.7	-11.7
Aldh6a1	1448104_at	aldehyde dehydrogenase family 6, subfamily A1	1390.9	464.1	-3.0
Alox15	1420338_at	arachidonate 15-lipoxygenase	33.9	436.6	12.9
Alox5ap	1452016_at	arachidonate 5-lipoxygenase activating protein	396.6	1284.4	3.2
Angpt2	1448831_at	angiopoietin 2	179.5	638.9	3.6
Angptl4	1417130_s_at	angiopoietin-like 4	272.6	1761.4	6.5
Ank1	1419421_at	ankyrin 1, erythroid	516.2	172.4	-3.0
Anp32a	1450407_a_at	acidic (leucine-rich) nuclear phosphoprotein 32 family, member A	576.4	96.8	-6.0
Anxa10	1449426_a_at	annexin A10	13369.8	3628.6	-3.7
Anxa3	1460330_at	annexin A3	1972.4	8424.0	4.3
Anxa6	1415818_at	annexin A6	945.6	303.8	-3.1
Anxa8	1417732_at	annexin A8	1092.4	7958.5	7.3
Anxa9	1431554_a_at	annexin A9	4124.3	1130.2	-3.6
Aoc3	1449396_at	amine oxidase, copper containing 3	1557.6	134.8	-11.6
Aox4	1419215_at	aldehyde oxidase 4	7162.4	714.5	-10.0
Apaf1	1452870_at	apoptotic peptidase activating factor 1	161.6	546.1	3.4
Apoa1	1419232_a_at	apolipoprotein A-I	1640.1	380.5	-4.3
Apoa2	1417950_a_at	apolipoprotein A-II	630.5	83.3	-7.6
Apoh	1416677_at	apolipoprotein H	540.8	72.0	-7.5
Arc	1418687_at	activity regulated cytoskeletal-associated protein	67.1	632.9	9.4
Areg	1421134_at	amphiregulin	291.0	4526.7	15.6
Arg1	1419549_at	arginase, liver	1474.6	22149.4	15.0
Arhgap24	1424842_a_at	Rho GTPase activating protein 24	706.4	163.5	-4.3
Arhgap6	1451867_x_at	Rho GTPase activating protein 6	556.8	128.0	-4.3
Armcx3	1460359_at	armadillo repeat containing, X-linked 3	797.3	164.2	-4.9
Arntl2	1425931_a_at	aryl hydrocarbon receptor nuclear translocator-like 2	88.3	318.8	3.6
Art3	1452474_a_at	ADP-ribosyltransferase 3	2628.4	205.3	-12.8
Asb13	1449459_s_at	ankyrin repeat and SOCS box-containing 13	521.7	174.9	-3.0
Aspn	1416652_at	asporin	1267.2	167.9	-7.5
Atp1b1	1451152_a_at	ATPase, Na+/K+ transporting, beta 1 polypeptide	4856.0	1430.8	-3.4
Atp2a3	1421129_a_at	ATPase, Ca++ transporting, ubiquitous	1366.8	237.0	-5.8
Atp4a	1421286_a_at	ATPase, H+/K+ exchanging, gastric, alpha polypeptide	2423.5	782.6	-3.1
Atp4b	1448911_at	ATPase, H+/K+ exchanging, beta polypeptide	3882.2	1192.5	-3.3

Atp6v0e2	1448211_at	ATPase, H+ transporting, lysosomal V0 subunit E2	938.5	216.2	-4.3
Atp6v1c2	1430306_a_at	ATPase, H+ transporting, lysosomal V1 subunit C2	1957.2	513.2	-3.8
Aurka	1424511_at	aurora kinase A	173.8	604.7	3.5
Avil	1419148_at	advillin	1430.4	403.1	-3.5
B3galnt1	1418736_at	UDP-GalNAc:betaGlcNAc beta 1,3-galactosaminyltransferase, polypeptide 1	688.6	152.3	-4.5
Barx1	1423342_at	BarH-like homeobox 1	833.0	134.6	-6.2
Bche	1437863_at	butyrylcholinesterase	368.0	27.4	-13.4
Bckdhb	1427153_at	branched chain ketoacid dehydrogenase E1, beta polypeptide	2010.2	624.1	-3.2
Bdh1	1452257_at	3-hydroxybutyrate dehydrogenase, type 1	3372.0	770.3	-4.4
Bex1	1448595_a_at	brain expressed gene 1	321.0	92.1	-3.5
Bhlhe22	1418271_at	basic helix-loop-helix family, member e22	37.1	435.1	11.7
Bhlhe40	1418025_at	basic helix-loop-helix family, member e40	969.1	3059.2	3.2
Birc5	1424278_a_at	baculoviral IAP repeat-containing 5	222.9	1227.4	5.5
Bmp2	1423635_at	bone morphogenetic protein 2	1451.4	462.6	-3.1
Bmp4	1422912_at	bone morphogenetic protein 4	821.6	128.3	-6.4
Bnc1	1424890_at	basonuclin 1	225.3	1439.1	6.4
Bst1	1449453_at	bone marrow stromal cell antigen 1	87.0	446.1	5.1
Bub1	1424046_at	budding uninhibited by benzimidazoles 1 homolog (S. cerevisiae)	113.9	914.1	8.0
Bub1b	1416961_at	budding uninhibited by benzimidazoles 1 homolog, beta (S. cerevisiae)	140.0	538.3	3.8
Cachd1	1436030_at	cache domain containing 1	844.7	207.9	-4.1
Cadm4	1426263_at	cell adhesion molecule 4	336.8	1637.1	4.9
Calcb	1422639_at	calcitonin-related polypeptide, beta	72.0	6898.0	95.8
Calml4	1424713_at	calmodulin-like 4	712.3	178.7	-4.0
Camkk1	1418954_at	calcium/calmodulin-dependent protein kinase kinase 1, alpha	641.0	126.4	-5.1
Cap2	1450910_at	CAP, adenylate cyclase-associated protein, 2 (yeast)	325.3	41.4	-7.9
Capg	1450355_a_at	capping protein (actin filament), gelsolin-like	1053.6	4868.4	4.6
Capn1	1417229_at	calpain 1	335.8	75.1	-4.5
Car3	1449434_at	carbonic anhydrase 3	1289.8	204.0	-6.3
Cartpt	1422825_at	CART prepropeptide	532.9	97.1	-5.5
Casp14	1418748_at	caspase 14	4846.4	392.7	-12.3
Cbx6	1424407_s_at	chromobox homolog 6	1162.0	389.4	-3.0
Ccbl2	1455991_at	cysteine conjugate-beta lyase 2	638.9	161.0	-4.0
Ccdc99	1424971_at	coiled-coil domain containing 99	115.2	366.5	3.2
Ccl2	1420380_at	chemokine (C-C motif) ligand 2	84.1	1077.6	12.8
Ccl3	1419561_at	chemokine (C-C motif) ligand 3	17.0	487.6	28.7
Ccl8	1419684_at	chemokine (C-C motif) ligand 8	101.9	1368.6	13.4
Ccl9	1417936_at	chemokine (C-C motif) ligand 9	363.1	1441.5	4.0
Ccnb1	1419943_s_at	cyclin B1	220.8	758.6	3.4
Ccnd1	1417420_at	cyclin D1	1323.7	4946.3	3.7
Ccr1	1419609_at	chemokine (C-C motif) receptor 1	51.4	592.2	11.5
Ccrl2	1427736_a_at	chemokine (C-C motif) receptor-like 2	88.7	310.4	3.5
Cd14	1417268_at	CD14 antigen	408.0	3730.6	9.1
Cd300a	1435903_at	CD300A antigen	43.8	332.4	7.6
Cd38	1433741_at	CD38 antigen	169.0	599.9	3.5
Cd52	1460218_at	CD52 antigen	370.2	1281.3	3.5
Cd53	1448617_at	CD53 antigen	350.5	1802.0	5.1
Cd55	1460242_at	CD55 antigen	750.7	156.5	-4.8
Cd68	1449164_at	CD68 antigen	258.7	797.5	3.1
Cd84	1422875_at	CD84 antigen	69.2	427.5	6.2
Cdc20	1439377_x_at	cell division cycle 20 homolog (S. cerevisiae)	1270.6	4701.0	3.7
Cdc2a	1448314_at	cell division cycle 2 homolog A (S. pombe)	421.6	1925.9	4.6
Cdca3	1452040_a_at	cell division cycle associated 3	192.4	602.8	3.1
Cdh5	1433956_at	cadherin 5	81.6	338.0	4.1
Cdkn1c	1417649_at	cyclin-dependent kinase inhibitor 1C (P57)	385.7	124.9	-3.1
Cdkn2a	1450140_a_at	cyclin-dependent kinase inhibitor 2A	20.3	312.4	15.4
Cdkn2b	1449152_at	cyclin-dependent kinase inhibitor 2B (p15, inhibits CDK4)	221.8	1447.1	6.5
Cdsn	1435191_at	corneodesmosin	5323.8	959.8	-5.5
Ceacam1	1425538_x_at	carcinoembryonic antigen-related cell adhesion molecule 1	235.8	3813.6	16.2
Cela1	1423693_at	chymotrypsin-like elastase family, member 1	4573.7	447.0	-10.2
Cenpa	1450842_a_at	centromere protein A	201.7	991.6	4.9
Cep55	1452242_at	centrosomal protein 55	139.0	766.0	5.5
Ces3	1435370_a_at	carboxylesterase 3	2775.9	129.1	-21.5
Cfd	1417867_at	complement factor D (adipsin)	310.2	90.7	-3.4
Cfl2	1418066_at	cofilin 2, muscle	1337.7	376.3	-3.6
Chchd10	1436990_s_at	coiled-coil-helix-coiled-coil-helix domain containing 10	7132.4	2192.5	-3.3
Chchd5	1451505_at	coiled-coil-helix-coiled-coil-helix domain containing 5	218.9	723.5	3.3
Chgb	1415885_at	chromogranin B	1117.8	80.5	-13.9
Chi3l1	1451537_at	chitinase 3-like 1	70.6	712.2	10.1
Chi3l3	1419764_at	chitinase 3-like 3	22.4	986.3	44.1
Chi3l4	1425450_at	chitinase 3-like 4	476.3	7236.1	15.2
Chodl	1451440_at	chondrolectin	852.8	178.2	-4.8
Chpt1	1455901_at	choline phosphotransferase 1	587.5	141.1	-4.2

Chrdl1	1421295_at	chordin-like 1	374.7	50.0	-7.5
Cideb	1418976_s_at	cell death-inducing DNA fragmentation factor, alpha subunit-like effector B	331.2	92.6	-3.6
Cited4	1425400_a_at	Cbp/p300-interacting transactivator, with Glu/Asp-rich carboxy-terminal domain, 4	1173.2	116.9	-10.0
Ckap2	1434748_at	cytoskeleton associated protein 2	242.1	756.6	3.1
Ckb	1455106_a_at	creatine kinase, brain	4668.1	594.5	-7.9
Cks1b	1416698_a_at	CDC28 protein kinase 1b	1867.6	5857.1	3.1
Cks2	1417457_at	CDC28 protein kinase regulatory subunit 2	687.7	2105.3	3.1
Clca3	1416306_at	chloride channel calcium activated 3	2163.8	77.3	-28.0
Cldn18	1449428_at	claudin 18	3103.4	433.9	-7.2
Cldn2	1417231_at	claudin 2	572.0	127.4	-4.5
Cldn23	1424409_at	claudin 23	2499.9	607.7	-4.1
Cldn3	1426332_a_at	claudin 3	38.9	300.4	7.7
Cldn7	1448393_at	claudin 7	177.9	1393.1	7.8
Cldn8	1449091_at	claudin 8	22.3	2811.3	126.1
Clec3b	1449466_at	C-type lectin domain family 3, member b	1362.5	186.2	-7.3
Clec4d	1420804_s_at	C-type lectin domain family 4, member d	24.9	486.3	19.5
Clec4e	1420330_at	C-type lectin domain family 4, member e	12.6	511.2	40.5
Clec4n	1425951_a_at	C-type lectin domain family 4, member n	30.9	841.9	27.2
Clec7a	1420699_at	C-type lectin domain family 7, member a	76.0	890.8	11.7
Cnn1	1417917_at	calponin 1	11427.9	627.2	-18.2
Col14a1	1427168_a_at	collagen, type XIV, alpha 1	2225.6	267.5	-8.3
Col17a1	1418799_a_at	collagen, type XVII, alpha 1	1170.0	4868.9	4.2
Col18a1	1418237_s_at	collagen, type XVIII, alpha 1	417.7	2177.7	5.2
Col4a5	1425476_at	collagen, type IV, alpha 5	3744.3	308.1	-12.2
Col4a6	1421007_at	collagen, type IV, alpha 6	623.7	64.7	-9.6
Col6a1	1448590_at	collagen, type VI, alpha 1	3390.0	774.4	-4.4
Col6a2	1452250_a_at	collagen, type VI, alpha 2	3746.8	1015.4	-3.7
Cpn1	1417745_at	carboxypeptidase N, polypeptide 1	313.3	105.2	-3.0
Cpox	1422492_at	coproporphyrinogen oxidase	562.1	181.0	-3.1
Crip2	1417311_at	cysteine rich protein 2	5030.3	1367.3	-3.7
Crisp1	1416325_at	cysteine-rich secretory protein 1	17.7	715.1	40.4
Cryab	1416455_a_at	crystallin, alpha B	1433.3	363.0	-3.9
Cryl1	1416795_at	crystallin, lambda 1	488.3	155.6	-3.1
Csf2rb	1421326_at	colony stimulating factor 2 receptor, beta, low-affinity (granulocyte-macrophage)	56.1	367.2	6.6
Csf3	1419427_at	colony stimulating factor 3 (granulocyte)	24.3	361.9	14.9
Cspg4	1423341_at	chondroitin sulfate proteoglycan 4	397.3	115.1	-3.5
Csrp1	1425811_a_at	cysteine and glycine-rich protein 1	6649.5	718.2	-9.3
Ctla2a	1448471_a_at	cytotoxic T lymphocyte-associated protein 2 alpha	667.6	3326.6	5.0
Ctsc	1416382_at	cathepsin C	2367.9	8863.0	3.7
Ctse	1418989_at	cathepsin E	15476.1	2629.4	-5.9
Ctsh	1418365_at	cathepsin H	634.1	2584.8	4.1
Ctss	1448591_at	cathepsin S	1295.6	6903.4	5.3
Cx3cl1	1415803_at	chemokine (C-X3-C motif) ligand 1	128.2	378.3	3.0
Cxcl1	1419209_at	chemokine (C-X-C motif) ligand 1	20.0	2004.9	100.0
Cxcl13	1417851_at	chemokine (C-X-C motif) ligand 13	239.0	979.1	4.1
Cxcl14	1418457_at	chemokine (C-X-C motif) ligand 14	58.6	324.8	5.5
Cxcl16	1449195_s_at	chemokine (C-X-C motif) ligand 16	115.0	664.3	5.8
Cxcl2	1449984_at	chemokine (C-X-C motif) ligand 2	16.0	1745.1	109.2
Cxcl5	1419728_at	chemokine (C-X-C motif) ligand 5	12.2	1774.0	145.6
Cxcl9	1418652_at	chemokine (C-X-C motif) ligand 9	39.6	525.5	13.3
Cyp2b10	1425645_s_at	cytochrome P450, family 2, subfamily b, polypeptide 10	100.6	576.4	5.7
Cyp2c65	1429994_s_at	cytochrome P450, family 2, subfamily c, polypeptide 65	1523.7	345.6	-4.4
Cyp2e1	1415994_at	cytochrome P450, family 2, subfamily e, polypeptide 1	646.8	41.8	-15.5
Cyp2s1	1428283_at	cytochrome P450, family 2, subfamily s, polypeptide 1	10378.4	2815.4	-3.7
Cyp3a11	1416809_at	cytochrome P450, family 3, subfamily a, polypeptide 11	497.9	19.3	-25.9
Cyp7b1	1421074_at	cytochrome P450, family 7, subfamily b, polypeptide 1	93.2	407.1	4.4
Cyr61	1416039_x_at	cysteine rich protein 61	332.3	1102.9	3.3
Cytip	1435697_a_at	cytohesin 1 interacting protein	250.5	813.2	3.2
Dab1	1427308_at	disabled homolog 1 (Drosophila)	364.0	33.1	-11.0
Dbf4	1418334_at	DBF4 homolog (S. cerevisiae)	387.5	1144.8	3.0
Dbp	1438211_s_at	D site albumin promoter binding protein	5067.3	728.8	-7.0
Dbt	1449118_at	dihydrolipoamide branched chain transacylase E2	621.9	205.4	-3.0
Ddr2	1422738_at	discoidin domain receptor family, member 2	531.4	99.5	-5.3
Ddx59	1460333_at	DEAD (Asp-Glu-Ala-Asp) box polypeptide 59	332.8	1042.7	3.1
Defb3	1421806_at	defensin beta 3	104.2	724.9	7.0
Defb4	1419600_at	defensin beta 4	44.1	636.5	14.4
Depdc7	1424303_at	DEP domain containing 7	132.3	610.7	4.6
Des	1426731_at	desmin	5379.5	562.3	-9.6
Dexi	1460174_at	dexamethasone-induced transcript	942.8	317.1	-3.0
Dkkl1	1417787_at	dickkopf-like 1	1454.3	303.9	-4.8
Dlg2	1421199_at	discs, large homolog 2 (Drosophila)	706.5	145.2	-4.9
Dlx3	1450475_at	distal-less homeobox 3	617.6	182.6	-3.4

Dmd	1448665_at	dystrophin, muscular dystrophy	463.9	62.6	-7.4
Dmpk	1434944_at	dystrophia myotonica-protein kinase	1285.5	89.0	-14.4
Dnajb5	1450436_s_at	DnaJ (Hsp40) homolog, subfamily B, member 5	610.4	63.8	-9.6
Dnajb6	1429776_a_at	DnaJ (Hsp40) homolog, subfamily B, member 6	292.9	868.6	3.0
Dnase1l2	1450936_a_at	deoxyribonuclease 1-like 2	3133.7	501.1	-6.3
Dnm2	1432004_a_at	dynamin 2	421.0	126.9	-3.3
Dpt	1418511_at	dermatopontin	3975.0	499.9	-8.0
Dram1	1424524_at	DNA-damage regulated autophagy modulator 1	210.4	898.8	4.3
Dsc1	1421460_at	desmocollin 1	2922.8	789.8	-3.7
Dsc2	1426911_at	desmocollin 2	1926.7	7934.7	4.1
Dst	1423626_at	dystonin	987.5	289.9	-3.4
Dtna	1429768_at	dystrobrevin alpha	476.9	37.2	-12.8
Dusp6	1415834_at	dual specificity phosphatase 6	304.4	4674.6	15.4
Dusp7	1460393_a_at	dual specificity phosphatase 7	88.7	355.9	4.0
Ect2	1419513_a_at	ect2 oncogene	122.2	433.8	3.5
Egfl6	1419332_at	EGF-like-domain, multiple 6	2291.3	415.9	-5.5
Elk3	1448797_at	ELK3, member of ETS oncogene family	330.2	1461.8	4.4
Elovl6	1417404_at	ELOVL family member 6, elongation of long chain fatty acids (yeast)	7991.9	1455.7	-5.5
Emb	1415857_at	embigin	478.3	3256.7	6.8
Emilin1	1416414_at	elastin microfibril interfacer 1	858.2	215.1	-4.0
Eno3	1417951_at	enolase 3, beta muscle	1038.7	319.8	-3.2
Enpep	1448649_at	glutamyl aminopeptidase	920.4	119.9	-7.7
Enpp1	1459546_s_at	ectonucleotide pyrophosphatase/phosphodiesterase 1	173.6	626.2	3.6
Enpp2	1448136_at	ectonucleotide pyrophosphatase/phosphodiesterase 2	1758.0	286.0	-6.1
Epcam	1416579_a_at	epithelial cell adhesion molecule	2095.9	7965.9	3.8
Epdr1	1450380_at	ependymin related protein 1 (zebrafish)	722.3	145.3	-5.0
Epgn	1449994_at	epithelial mitogen	88.6	1871.1	21.1
Epha2	1421151_a_at	Eph receptor A2	190.5	1219.9	6.4
Eppk1	1427537_at	epiplakin 1	3884.1	966.8	-4.0
Eps8l1	1431821_a_at	EPS8-like 1	3124.0	803.9	-3.9
Ero1l	1419029_at	ERO1-like (S. cerevisiae)	1105.5	4076.2	3.7
Ethe1	1417203_at	ethylmalonic encephalopathy 1	2978.5	944.0	-3.2
Ets1	1452163_at	E26 avian leukemia oncogene 1, 5' domain	206.6	619.9	3.0
Evi2a	1450241_a_at	ecotropic viral integration site 2a	117.6	393.5	3.3
Expi	1417160_s_at	extracellular proteinase inhibitor	72.7	346.3	4.8
Eya2	1424127_at	eyes absent 2 homolog (Drosophila)	93.8	433.6	4.6
Fabp1	1448764_a_at	fatty acid binding protein 1, liver	846.0	138.0	-6.1
Fabp2	1418438_at	fatty acid binding protein 2, intestinal	1999.0	331.7	-6.0
Fam129a	1422567_at	family with sequence similarity 129, member A	1842.9	421.3	-4.4
Fam13c	1448557_at	family with sequence similarity 13, member C	322.7	61.7	-5.2
Fam162a	1451385_at	family with sequence similarity 162, member A	3845.6	16413.3	4.3
Fam26e	1424680_at	family with sequence similarity 26, member E	407.2	57.7	-7.1
Fam60a	1448126_at	family with sequence similarity 60, member A	138.4	683.4	4.9
Fbln2	1423407_a_at	fibulin 2	357.6	1097.4	3.1
Fbp2	1449088_at	fructose bisphosphatase 2	1042.8	3291.3	3.2
Fbxo32	1448747_at	F-box protein 32	2382.6	698.9	-3.4
Fcer1g	1418340_at	Fc receptor, IgE, high affinity I, gamma polypeptide	183.0	867.0	4.7
Fcgbp	1426872_at	Fc fragment of IgG binding protein	652.8	210.7	-3.1
Fcgr1	1417876_at	Fc receptor, IgG, high affinity I	47.1	327.0	6.9
Fcgr2b	1435477_s_at	Fc receptor, IgG, low affinity IIb	310.7	1580.8	5.1
Fcgr3	1448620_at	Fc receptor, IgG, low affinity III	455.9	2571.8	5.6
Fermt2	1434180_at	fermitin family homolog 2 (Drosophila)	4140.4	769.6	-5.4
Fermt3	1433963_a_at	fermitin family homolog 3 (Drosophila)	817.8	240.3	-3.4
Fes	1452410_a_at	feline sarcoma oncogene	113.7	453.3	4.0
Fetub	1449555_a_at	fetuin beta	5683.2	843.0	-6.7
Fgf22	1460296_a_at	fibroblast growth factor 22	366.4	105.6	-3.5
Fgfr2	1433489_s_at	fibroblast growth factor receptor 2	2350.3	532.5	-4.4
Fgl2	1421855_at	fibrinogen-like protein 2	1921.8	318.7	-6.0
Fhl1	1417872_at	four and a half LIM domains 1	12740.9	1706.1	-7.5
Fibin	1419376_at	fin bud initiation factor homolog (zebrafish)	670.9	189.9	-3.5
Fignl1	1422430_at	fidgetin-like 1	125.3	387.0	3.1
Flg	1427268_at	filaggrin	8181.3	1334.0	-6.1
Flna	1426677_at	filamin, alpha	9958.7	2663.7	-3.7
Flnc	1449073_at	filamin C, gamma	430.6	139.2	-3.1
Fmo1	1417429_at	flavin containing monooxygenase 1	376.3	66.3	-5.7
Fmo2	1422905_s_at	flavin containing monooxygenase 2	870.4	135.4	-6.4
Fnbp1	1426983_at	formin binding protein 1	824.2	257.1	-3.2
Fndc1	1453321_at	fibronectin type III domain containing 1	767.9	216.9	-3.5
Fosl1	1417487_at	fos-like antigen 1	84.7	626.2	7.4
Foxa2	1422833_at	forkhead box A2	359.8	79.7	-4.5
Foxa3	1431900_a_at	forkhead box A3	342.2	89.5	-3.8
Foxc1	1419486_at	forkhead box C1	48.1	516.9	10.7

Foxg1	1418357_at	forkhead box G1	10.9	595.7	54.6
Fpr2	1422953_at	formyl peptide receptor 2	25.6	598.9	23.4
Fscn1	1416514_a_at	fascin homolog 1, actin bundling protein (Strongylocentrotus purpuratus)	1851.4	6288.7	3.4
Fst	1421365_at	follistatin	297.6	5370.1	18.0
Fxyd1	1421374_a_at	FXYD domain-containing ion transport regulator 1	1714.6	192.7	-8.9
Fxyd4	1418207_at	FXYD domain-containing ion transport regulator 4	54.3	763.7	14.1
Fxyd6	1417343_at	FXYD domain-containing ion transport regulator 6	904.2	186.4	-4.8
Fzd2	1418534_at	frizzled homolog 2 (Drosophila)	857.5	153.4	-5.6
Fzd4	1419301_at	frizzled homolog 4 (Drosophila)	794.2	214.6	-3.7
Gabrp	1424647_at	gamma-aminobutyric acid (GABA) A receptor, pi	54.7	440.4	8.0
Gal3st1	1454078_a_at	galactose-3-O-sulfotransferase 1	1477.2	218.8	-6.8
Gast	1422915_at	gastrin	6048.0	117.7	-51.4
Gc	1426547_at	group specific component	531.6	28.6	-18.6
Gda	1422868_s_at	guanine deaminase	65.9	587.3	8.9
Gfpt1	1449268_at	glutamine fructose-6-phosphate transaminase 1	93.0	404.1	4.3
Ggta1	1418483_a_at	glycoprotein galactosyltransferase alpha 1, 3	468.7	1563.4	3.3
Ghr	1417962_s_at	growth hormone receptor	2106.6	620.5	-3.4
Gjb1	1448767_s_at	gap junction protein, beta 1	729.9	144.6	-5.0
Gjb3	1416715_at	gap junction protein, beta 3	1721.6	363.4	-4.7
Gjb4	1422179_at	gap junction protein, beta 4	1015.1	329.9	-3.1
Gjb5	1449204_at	gap junction protein, beta 5	2146.5	270.0	-8.0
Gjc1	1449094_at	gap junction protein, gamma 1	463.8	119.7	-3.9
Gldc	1416049_at	glycine decarboxylase	1216.4	292.9	-4.2
Glycam1	1424825_a_at	glycosylation dependent cell adhesion molecule 1	1274.4	238.0	-5.4
Gnai1	1454959_s_at	guanine nucleotide binding protein (G protein), alpha inhibiting 1	5303.2	944.0	-5.6
Gp1bb	1422977_at	glycoprotein Ib, beta polypeptide	404.9	117.8	-3.4
Gpc3	1450990_at	glypican 3	449.3	91.4	-4.9
Gpld1	1418050_at	glycosylphosphatidylinositol specific phospholipase D1	3748.4	957.6	-3.9
Gpm6a	1456741_s_at	glycoprotein m6a	463.7	75.8	-6.1
Gpm6b	1425942_a_at	glycoprotein m6b	651.8	199.9	-3.3
Gpr137b-ps	1439256_x_at	G protein-coupled receptor 137B, pseudogene	536.8	1732.5	3.2
Gprc5b	1451411_at	G protein-coupled receptor, family C, group 5, member B	124.0	515.7	4.2
Grem2	1418492_at	gremlin 2 homolog, cysteine knot superfamily (Xenopus laevis)	1943.7	308.1	-6.3
Grp	1424525_at	gastrin releasing peptide	823.6	79.3	-10.4
Gsdma	1423634_at	gasdermin A	4191.5	730.5	-5.7
Gsn	1415812_at	gelsolin	14693.1	3444.7	-4.3
Gsr	1421817_at	glutathione reductase	714.2	3228.6	4.5
Gstm1	1448330_at	glutathione S-transferase, mu 1	8688.6	1284.9	-6.8
Gstm2	1416411_at	glutathione S-transferase, mu 2	2388.2	335.6	-7.1
Gstm3	1427474_s_at	glutathione S-transferase, mu 3	1125.7	133.1	-8.5
Gstm6	1422072_a_at	glutathione S-transferase, mu 6	684.7	185.5	-3.7
Gstt1	1418186_at	glutathione S-transferase, theta 1	945.4	181.8	-5.2
Gstt3	1423891_at	glutathione S-transferase, theta 3	642.9	194.1	-3.3
Gucy1b3	1420872_at	guanylate cyclase 1, soluble, beta 3	721.6	139.2	-5.2
H19	1448194_a_at	H19 fetal liver mRNA	2519.6	599.3	-4.2
Has2	1418678_at	hyaluronan synthase 2	39.7	371.0	9.3
Has3	1420589_at	hyaluronan synthase 3	96.9	920.2	9.5
Havcr2	1451584_at	hepatitis A virus cellular receptor 2	16.4	378.1	23.0
Hbegf	1418350_at	heparin-binding EGF-like growth factor	691.6	3186.4	4.6
Hdc	1454713_s_at	histidine decarboxylase	91.9	523.4	5.7
Hipk2	1424863_a_at	homeodomain interacting protein kinase 2	861.8	225.0	-3.8
Hist1h4i	1424854_at	histone cluster 1, H4i	136.3	421.3	3.1
Hk1	1420901_a_at	hexokinase 1	1704.9	523.6	-3.3
Hlf	1434736_at	hepatic leukemia factor	440.8	68.5	-6.4
Hmga1	1416184_s_at	high mobility group AT-hook 1	733.8	3990.0	5.4
Hmga2	1450780_s_at	high mobility group AT-hook 2	58.5	4402.1	75.2
Hmgb3	1416155_at	high mobility group box 3	318.9	1157.9	3.6
Hnf4a	1427001_s_at	hepatic nuclear factor 4, alpha	624.0	134.5	-4.6
Hnrnpl	1424101_at	heterogeneous nuclear ribonucleoprotein L	289.6	993.2	3.4
Homer2	1424367_a_at	homer homolog 2 (Drosophila)	1934.1	407.3	-4.7
Hoxa5	1448926_at	homeo box A5	300.7	56.9	-5.3
Hoxb6	1451660_a_at	homeo box B6	336.9	111.9	-3.0
Hoxc5	1450832_at	homeo box C5	508.8	121.9	-4.2
Hp	1448881_at	haptoglobin	220.7	1206.4	5.5
Hpgd	1419905_s_at	hydroxyprostaglandin dehydrogenase 15 (NAD)	8217.3	2656.9	-3.1
Hpx	1423944_at	hemopexin	439.7	83.4	-5.3
Hrnr	1451613_at	hornerin	10493.9	1550.9	-6.8
Hs3st1	1423450_a_at	heparan sulfate (glucosamine) 3-O-sulfotransferase 1	215.8	763.6	3.5
Hsd11b1	1449038_at	hydroxysteroid 11-beta dehydrogenase 1	2193.5	603.9	-3.6
Hsd17b11	1434642_at	hydroxysteroid (17-beta) dehydrogenase 11	4738.1	1490.9	-3.2
Hsd17b2	1418352_at	hydroxysteroid (17-beta) dehydrogenase 2	4823.2	1554.0	-3.1
Hspb2	1429888_a_at	heat shock protein 2	355.7	105.8	-3.4
Hspb7	1434927_at	heat shock protein family, member 7 (cardiovascular)	933.8	107.3	-8.7

Hspb8	1417013_at	heat shock protein 8	2806.9	751.9	-3.7
Htra3	1419292_at	HtrA serine peptidase 3	825.2	205.0	-4.0
Icam1	1424067_at	intercellular adhesion molecule 1	172.6	687.0	4.0
Id4	1438441_at	inhibitor of DNA binding 4	317.7	43.9	-7.2
Iffo2	1426597_s_at	intermediate filament family orphan 2	157.6	584.3	3.7
Ifi202b	1421551_s_at	interferon activated gene 202B	335.3	4164.2	12.4
Ifi204	1419603_at	interferon activated gene 204	42.4	354.4	8.4
Ifitm6	1440865_at	interferon induced transmembrane protein 6	38.8	363.0	9.3
Igf2bp2	1437103_at	insulin-like growth factor 2 mRNA binding protein 2	112.0	952.0	8.5
Igfbp2	1454159_a_at	insulin-like growth factor binding protein 2	3635.9	483.7	-7.5
Igfbp3	1423062_at	insulin-like growth factor binding protein 3	365.7	8666.4	23.7
Igfbp5	1452114_s_at	insulin-like growth factor binding protein 5	3511.1	744.8	-4.7
Igfbp6	1417933_at	insulin-like growth factor binding protein 6	1511.3	199.5	-7.6
Igj	1424305_at	immunoglobulin joining chain	108.3	2774.1	25.6
Igl-V1	1430523_s_at	immunoglobulin lambda chain, variable 1	22.4	434.7	19.4
Igsf8	1460675_at	immunoglobulin superfamily, member 8	291.4	960.2	3.3
Il13ra1	1427164_at	interleukin 13 receptor, alpha 1	297.2	907.2	3.1
Il17b	1431693_a_at	interleukin 17B	2044.6	148.7	-13.7
Il18	1417932_at	interleukin 18	9188.7	2064.1	-4.5
Il1a	1421473_at	interleukin 1 alpha	86.6	489.7	5.7
Il1b	1449399_a_at	interleukin 1 beta	75.1	1569.4	20.9
Il1f5	1421370_a_at	interleukin 1 family, member 5 (delta)	12328.2	2197.7	-5.6
Il1f8	1425715_at	interleukin 1 family, member 8	3594.4	824.7	-4.4
Il1r1	1448950_at	interleukin 1 receptor, type I	249.2	1007.4	4.0
Il24	1426181_a_at	interleukin 24	26.3	623.0	23.7
Il4ra	1421034_a_at	interleukin 4 receptor, alpha	261.3	857.1	3.3
Il6	1450297_at	interleukin 6	12.2	479.1	39.4
Il6st	1460295_s_at	interleukin 6 signal transducer	456.5	123.5	-3.7
Incenp	1439436_x_at	inner centromere protein	615.0	1920.6	3.1
Isg20	1419569_a_at	interferon-stimulated protein	429.3	1589.8	3.7
Isl1	1450723_at	ISL1 transcription factor, LIM/homeodomain	157.9	477.2	3.0
Islr	1418450_at	immunoglobulin superfamily containing leucine-rich repeat	1189.7	228.8	-5.2
Itga6	1422445_at	integrin alpha 6	1327.7	3942.3	3.0
Itga7	1418393_a_at	integrin alpha 7	322.3	88.9	-3.6
Itga9	1460285_at	integrin alpha 9	723.6	185.1	-3.9
Itgb2	1450678_at	integrin beta 2	172.5	846.9	4.9
Itih5	1429159_at	inter-alpha (globulin) inhibitor H5	4824.4	795.2	-6.1
Iyd	1451547_at	iodotyrosine deiodinase	480.6	153.4	-3.1
Kcnmb1	1421400_at	potassium large conductance calcium-activated channel, subfamily M, beta member 1	585.4	80.7	-7.3
Kcnn4	1435945_a_at	potassium intermediate/small conductance calcium-activated channel, subfamily N, member 4	629.8	3143.5	5.0
Kif20a	1449207_a_at	kinesin family member 20A	101.3	389.4	3.8
Kif23	1455990_at	kinesin family member 23	125.2	472.8	3.8
Klf15	1448181_at	Kruppel-like factor 15	383.1	96.2	-4.0
Klf9	1456341_a_at	Kruppel-like factor 9	2064.3	397.9	-5.2
Klhl13	1448269_a_at	kelch-like 13 (Drosophila)	383.1	84.8	-4.5
Klk6	1448982_at	kallikrein related-peptidase 6	1047.3	3665.3	3.5
Klra18	1426127_x_at	killer cell lectin-like receptor, subfamily A, member 18	310.5	4970.8	16.0
Krt1	1422481_at	keratin 1	27917.1	8818.3	-3.2
Krt16	1448932_at	keratin 16	86.8	10070.2	116.0
Krt17	1423227_at	keratin 17	227.1	3088.4	13.6
Krt20	1426284_at	keratin 20	1764.8	312.1	-5.7
Krt4	1418735_at	keratin 4	24449.9	7892.0	-3.1
Krt6a	1422784_at	keratin 6A	241.2	20785.6	86.2
Krt6b	1422588_at	keratin 6B	2756.1	26108.4	9.5
Krt7	1423952_a_at	keratin 7	1225.9	7339.7	6.0
Krt8	1420647_a_at	keratin 8	16262.0	5042.4	-3.2
Lama2	1426285_at	laminin, alpha 2	469.7	100.0	-4.7
Lama3	1427512_a_at	laminin, alpha 3	364.0	4147.6	11.4
Lamb2	1416513_at	laminin, beta 2	591.3	175.4	-3.4
Lamb3	1417812_a_at	laminin, beta 3	510.0	2377.1	4.7
Lamc2	1421279_at	laminin, gamma 2	544.3	3160.9	5.8
Laptm5	1436905_x_at	lysosomal-associated protein transmembrane 5	564.3	1863.6	3.3
Lce1a1	1420676_at	late cornified envelope 1A1	15801.0	3411.6	-4.6
Lce1a2	1420350_at	late cornified envelope 1A2	18579.5	5080.5	-3.7
Lce1b	1419409_at	late cornified envelope 1B	8715.4	1845.1	-4.7
Lce1g	1421316_at	late cornified envelope 1G	312.3	77.5	-4.0
Lce1h	1449959_x_at	late cornified envelope 1H	20812.5	4605.1	-4.5
Lce1i	1420741_x_at	late cornified envelope 1I	25792.0	6775.4	-3.8
Lce1l	1418855_at	late cornified envelope 1L	1186.9	192.0	-6.2
Lcn2	1427747_a_at	lipocalin 2	23.1	651.7	28.3
Ldb2	1421101_a_at	LIM domain binding 2	643.0	171.9	-3.7
Lgals2	1417079_s_at	lectin, galactose-binding, soluble 2	4724.2	957.7	-4.9

Lgals4	1451336_at	lectin, galactose binding, soluble 4	2691.5	454.3	-5.9
Lgals7	1424594_at	lectin, galactose binding, soluble 7	2224.7	386.1	-5.8
Lims2	1424408_at	LIM and senescent cell antigen like domains 2	2764.3	248.6	-11.1
Lmod1	1427485_at	leiomodin 1 (smooth muscle)	550.9	93.8	-5.9
Lnx1	1455825_s_at	ligand of numb-protein X 1	2248.2	492.9	-4.6
Lox	1416121_at	lysyl oxidase	464.3	1606.2	3.5
Lpar1	1448606_at	lysophosphatidic acid receptor 1	1303.3	318.4	-4.1
Lpcat1	1424459_at	lysophosphatidylcholine acyltransferase 1	202.3	683.5	3.4
Lphn1	1428510_at	latrophilin 1	443.0	105.8	-4.2
Lpp	1454899_at	LIM domain containing preferred translocation partner in lipoma	4363.3	1281.0	-3.4
Lst1	1425548_a_at	leukocyte specific transcript 1	102.9	456.9	4.4
Ltbp2	1418061_at	latent transforming growth factor beta binding protein 2	30.6	337.9	11.0
Ltbp4	1436665_a_at	latent transforming growth factor beta binding protein 4	1323.8	418.5	-3.2
Ltf	1450009_at	lactotransferrin	163.3	21298.5	130.5
Lxn	1416503_at	latexin	733.0	3203.2	4.4
Ly6g6c	1422749_at	lymphocyte antigen 6 complex, locus G6C	11343.0	2714.6	-4.2
Ly86	1422903_at	lymphocyte antigen 86	157.6	631.8	4.0
Lyz2	1423547_at	lysozyme 2	2122.7	8302.3	3.9
Macrod1	1419687_at	MACRO domain containing 1	565.8	179.4	-3.2
Mad2l1	1422460_at	MAD2 mitotic arrest deficient-like 1 (yeast)	348.6	1312.0	3.8
Mafb	1451716_at	v-maf musculoaponeurotic fibrosarcoma oncogene family, protein B (avian)	210.4	626.0	3.0
Mansc1	1423284_at	MANSC domain containing 1	1204.6	249.0	-4.8
Map1lc3a	1451290_at	microtubule-associated protein 1 light chain 3 alpha	1241.2	419.9	-3.0
Mapre2	1451989_a_at	microtubule-associated protein, RP/EB family, member 2	2834.2	600.2	-4.7
Marveld1	1439381_x_at	MARVEL (membrane-associating) domain containing 1	8259.6	1750.8	-4.7
Matn2	1419442_at	matrilin 2	593.8	136.5	-4.4
Mb	1451203_at	myoglobin	64.3	609.1	9.5
Mbnl3	1422836_at	muscleblind-like 3 (Drosophila)	108.6	465.0	4.3
Mboat2	1425029_a_at	membrane bound O-acyltransferase domain containing 2	5457.6	1454.0	-3.8
Me2	1426573_at	malic enzyme 2, NAD(+)-dependent, mitochondrial	445.9	127.1	-3.5
Melk	1416558_at	maternal embryonic leucine zipper kinase	171.2	526.0	3.1
Mgll	1426785_s_at	monoglyceride lipase	2072.8	516.2	-4.0
Mid2	1422216_at	midline 2	464.4	152.2	-3.1
Mmp10	1420450_at	matrix metallopeptidase 10	31.6	498.1	15.8
Mmp12	1449153_at	matrix metallopeptidase 12	17.5	1820.9	104.1
Mmp13	1417256_at	matrix metallopeptidase 13	14.8	927.5	62.5
Mmp3	1418945_at	matrix metallopeptidase 3	51.8	483.2	9.3
Mmp9	1448291_at	matrix metallopeptidase 9	73.6	442.2	6.0
Morc4	1434436_at	microrchidia 4	179.2	713.7	4.0
Morf4l1	1437801_at	mortality factor 4 like 1	1317.7	434.6	-3.0
Moxd1	1422643_at	monooxygenase, DBH-like 1	196.6	3079.9	15.7
Mpeg1	1427076_at	macrophage expressed gene 1	294.3	1206.5	4.1
Mrgprf	1425894_at	MAS-related GPR, member F	581.2	66.7	-8.7
Mrvi1	1422245_a_at	MRV integration site 1	432.2	35.0	-12.3
Ms4a6b	1418826_at	membrane-spanning 4-domains, subfamily A, member 6B	209.7	669.3	3.2
Ms4a6d	1419599_s_at	membrane-spanning 4-domains, subfamily A, member 6D	91.0	960.8	10.6
Ms4a7	1424754_at	membrane-spanning 4-domains, subfamily A, member 7	135.5	615.6	4.5
Mt4	1450645_at	metallothionein 4	19576.1	6329.1	-3.1
Mtm1	1434278_at	X-linked myotubular myopathy gene 1	426.0	1850.7	4.3
Muc5ac	1430899_at	mucin 5, subtypes A and C, tracheobronchial/gastric	11100.2	668.6	-16.6
Mustn1	1427201_at	musculoskeletal, embryonic nuclear protein 1	573.0	138.1	-4.1
Mxra8	1452330_a_at	matrix-remodelling associated 8	2241.2	595.5	-3.8
Myh11	1448962_at	myosin, heavy polypeptide 11, smooth muscle	10900.9	795.0	-13.7
Myh9	1420170_at	myosin, heavy polypeptide 9, non-muscle	265.4	854.3	3.2
Myl1	1452651_a_at	myosin, light polypeptide 1	34.9	412.8	11.8
Myl9	1452670_at	myosin, light polypeptide 9, regulatory	20264.2	1625.2	-12.5
Mylk	1425506_at	myosin, light polypeptide kinase	10372.5	1394.5	-7.4
Mylpf	1448371_at	myosin light chain, phosphorylatable, fast skeletal muscle	72.6	440.8	6.1
Myo1c	1449551_at	myosin IC	693.1	221.1	-3.1
Myo5c	1424933_at	myosin VC	606.9	182.2	-3.3
Myof	1427318_s_at	myoferlin	628.0	2978.6	4.7
Myom1	1420693_at	myomesin 1	361.5	57.3	-6.3
Ncald	1417569_at	neurocalcin delta	608.6	196.2	-3.1
Ncf4	1418465_at	neutrophil cytosolic factor 4	105.4	539.3	5.1
Ndn	1435382_at	necdin	1699.0	175.1	-9.7
Ndrg1	1456174_x_at	N-myc downstream regulated gene 1	2059.4	7732.4	3.8
Ndrg4	1426615_s_at	N-myc downstream regulated gene 4	1450.5	321.9	-4.5
Nfix	1436363_a_at	nuclear factor I/X	4120.9	1193.2	-3.5
Ngb	1417996_at	neuroglobin	411.4	98.7	-4.2
Nkd1	1417278_a_at	naked cuticle 1 homolog (Drosophila)	393.8	52.7	-7.5
Nmu	1419325_at	neuromedin U	6168.9	1301.5	-4.7
Npnt	1452106_at	nephronectin	2579.2	267.6	-9.6

Npr2	1427191_at	natriuretic peptide receptor 2	373.9	94.8	-3.9
Nqo1	1423627_at	NAD(P)H dehydrogenase, quinone 1	3244.8	917.3	-3.5
Nrbp2	1424544_at	nuclear receptor binding protein 2	687.9	207.9	-3.3
Nrd1	1424391_at	nardilysin, N-arginine dibasic convertase, NRD convertase 1	4331.9	1405.6	-3.1
Nt5dc2	1424882_a_at	5'-nucleotidase domain containing 2	90.0	652.0	7.2
Ntn1	1454974_at	netrin 1	561.6	108.4	-5.2
Nupr1	1419665_a_at	nuclear protein 1	1129.7	5450.4	4.8
Odc1	1427364_a_at	ornithine decarboxylase, structural 1	551.7	1798.0	3.3
Ogn	1419663_at	osteoglycin	6092.0	403.9	-15.1
Optc	1420578_at	opticin	382.0	31.6	-12.1
Osbpl1a	1460192_at	oxysterol binding protein-like 1A	2835.9	566.8	-5.0
Otub2	1417575_at	OTU domain, ubiquitin aldehyde binding 2	2586.9	810.6	-3.2
P2ry14	1424733_at	purinergic receptor P2Y, G-protein coupled, 14	560.1	112.0	-5.0
P2ry2	1450318_a_at	purinergic receptor P2Y, G-protein coupled 2	81.4	307.3	3.8
Padi1	1419323_at	peptidyl arginine deiminase, type I	204.1	941.2	4.6
Padi3	1419767_at	peptidyl arginine deiminase, type III	22.2	632.1	28.5
Padi4	1422760_at	peptidyl arginine deiminase, type IV	35.6	347.8	9.8
Pah	1454638_a_at	phenylalanine hydroxylase	358.9	20.9	-17.2
Pak1	1420979_at	p21 protein (Cdc42/Rac)-activated kinase 1	161.0	616.4	3.8
Parp8	1451474_a_at	poly (ADP-ribose) polymerase family, member 8	149.5	614.9	4.1
Pbk	1448627_s_at	PDZ binding kinase	441.9	1362.0	3.1
Pcdh7	1449249_at	protocadherin 7	114.3	900.4	7.9
Pcolce	1437165_a_at	procollagen C-endopeptidase enhancer protein	3756.7	839.7	-4.5
Pcolce2	1451527_at	procollagen C-endopeptidase enhancer 2	636.4	137.1	-4.6
Pcp4	1460214_at	Purkinje cell protein 4	313.1	52.5	-6.0
Pcp4l1	1452913_at	Purkinje cell protein 4-like 1	457.5	44.4	-10.3
Pcsk6	1426981_at	proprotein convertase subtilisin/kexin type 6	1712.4	498.3	-3.4
Pcx	1416383_a_at	pyruvate carboxylase	986.5	283.5	-3.5
Pdlim3	1449178_at	PDZ and LIM domain 3	4250.0	327.6	-13.0
Pdlim4	1417928_at	PDZ and LIM domain 4	704.4	161.5	-4.4
Pdlim5	1429783_at	PDZ and LIM domain 5	101.9	404.0	4.0
Pdzrn3	1416846_a_at	PDZ domain containing RING finger 3	1960.4	396.3	-4.9
Peg3	1417355_at	paternally expressed 3	571.5	81.2	-7.0
Penk	1427038_at	preproenkephalin	777.9	226.7	-3.4
Pfkfb3	1416432_at	6-phosphofructo-2-kinase/fructose-2,6-biphosphatase 3	146.4	454.3	3.1
Pga5	1421113_at	pepsinogen 5, group I	733.1	177.6	-4.1
Pgc	1415786_at	progastricsin (pepsinogen C)	1236.8	286.3	-4.3
Pgcp	1416441_at	plasma glutamate carboxypeptidase	860.1	251.5	-3.4
Pgf	1418471_at	placental growth factor	100.6	309.2	3.1
Phlda1	1418835_at	pleckstrin homology-like domain, family A, member 1	1017.9	4029.1	4.0
Pi15	1421403_at	peptidase inhibitor 15	76.2	437.2	5.7
Pi16	1453839_a_at	peptidase inhibitor 16	953.0	221.5	-4.3
Pitpnm2	1419757_at	phosphatidylinositol transfer protein, membrane-associated 2	318.2	73.4	-4.3
Pitx2	1424797_a_at	paired-like homeodomain transcription factor 2	709.8	83.0	-8.6
Pkdcc	1454838_s_at	protein kinase domain containing, cytoplasmic	1589.7	159.4	-10.0
Pla1a	1417785_at	phospholipase A1 member A	84.6	373.4	4.4
Pla2g10	1451502_at	phospholipase A2, group X	1314.9	327.7	-4.0
Pla2g12b	1419614_at	phospholipase A2, group XIIB	377.1	68.4	-5.5
Plagl1	1426208_x_at	pleiomorphic adenoma gene-like 1	8841.7	1661.8	-5.3
Plat	1415806_at	plasminogen activator, tissue	298.1	7816.5	26.2
Plaur	1452521_a_at	plasminogen activator, urokinase receptor	219.9	1089.1	5.0
Plce1	1452398_at	phospholipase C, epsilon 1	484.8	98.1	-4.9
Plek	1448748_at	pleckstrin	90.5	718.9	7.9
Plekhh1	1435053_s_at	pleckstrin homology domain containing, family H (with MyTH4 domain) member 1	202.1	603.1	3.0
Plin2	1448318_at	perilipin 2	3418.2	12057.0	3.5
Plin4	1418595_at	perilipin 4	807.8	75.0	-10.8
Plk2	1427005_at	polo-like kinase 2 (Drosophila)	473.1	3409.4	7.2
Plk4	1419838_s_at	polo-like kinase 4 (Drosophila)	194.4	640.9	3.3
Pln	1460332_at	phospholamban	1264.7	64.0	-19.7
Pls1	1460406_at	plastin 1 (I-isoform)	1419.8	415.0	-3.4
Plxdc2	1418912_at	plexin domain containing 2	2073.8	646.7	-3.2
Pm20d1	1438980_x_at	peptidase M20 domain containing 1	307.9	87.7	-3.5
Pmaip1	1418203_at	phorbol-12-myristate-13-acetate-induced protein 1	136.7	3700.8	27.1
Pmp22	1417133_at	peripheral myelin protein 22	2383.7	530.4	-4.5
Pnck	1422711_a_at	pregnancy upregulated non-ubiquitously expressed CaM kinase	341.1	64.7	-5.3
Pnpla3	1420655_at	patatin-like phospholipase domain containing 3	415.9	130.9	-3.2
Popdc2	1417806_at	popeye domain containing 2	306.7	103.2	-3.0
Ppap2a	1422619_at	phosphatidic acid phosphatase type 2A	2114.2	678.4	-3.1
Ppara	1449051_at	peroxisome proliferator activated receptor alpha	1227.4	143.0	-8.6
Pparg	1420715_a_at	peroxisome proliferator activated receptor gamma	875.4	204.7	-4.3
Ppbp	1418480_at	pro-platelet basic protein	39.6	15481.6	391.2
Ppp1r14a	1418086_at	protein phosphatase 1, regulatory (inhibitor) subunit 14A	1713.9	83.0	-20.7

Ppp1r3c	1433691_at	protein phosphatase 1, regulatory (inhibitor) subunit 3C	1233.0	148.3	-8.3
Prc1	1423774_a_at	protein regulator of cytokinesis 1	138.2	534.6	3.9
Prc1	1423775_s_at	protein regulator of cytokinesis 1	258.3	901.1	3.5
Prelp	1416321_s_at	proline arginine-rich end leucine-rich repeat	1997.8	393.4	-5.1
Prkg1	1449876_at	protein kinase, cGMP-dependent, type I	412.6	53.5	-7.7
Prkg2	1435162_at	protein kinase, cGMP-dependent, type II	319.9	2767.2	8.6
Prss12	1420388_at	protease, serine, 12 neurotrypsin (motopsin)	136.5	889.0	6.5
Prss22	1420352_at	protease, serine, 22	76.5	2344.9	30.6
Psapl1	1429457_at	prosaposin-like 1	12245.0	3733.9	-3.3
Psd	1435780_at	pleckstrin and Sec7 domain containing	538.0	139.9	-3.8
Psors1c2	1420467_at	psoriasis susceptibility 1 candidate 2 (human)	492.6	145.3	-3.4
Psrc1	1417323_at	proline/serine-rich coiled-coil 1	87.7	308.9	3.5
Pstpip2	1455405_at	proline-serine-threonine phosphatase-interacting protein 2	126.6	611.3	4.8
Ptger3	1450344_a_at	prostaglandin E receptor 3 (subtype EP3)	601.9	136.3	-4.4
Ptges	1449450_at	prostaglandin E synthase	136.9	1559.1	11.4
Ptgis	1448816_at	prostaglandin I2 (prostacyclin) synthase	2020.9	361.1	-5.6
Ptgs1	1423414_at	prostaglandin-endoperoxide synthase 1	1635.4	471.5	-3.5
Ptgs2	1417262_at	prostaglandin-endoperoxide synthase 2	29.1	409.8	14.1
Pthlh	1422324_a_at	parathyroid hormone-like peptide	110.2	1716.8	15.6
Ptms	1428707_at	parathymosin	2883.2	928.7	-3.1
Ptn	1448254_at	pleiotrophin	1677.2	462.0	-3.6
Ptprc	1422124_a_at	protein tyrosine phosphatase, receptor type, C	243.8	753.0	3.1
Ptprz1	1427019_at	protein tyrosine phosphatase, receptor type Z, polypeptide 1	378.6	2221.9	5.9
Pttg1	1438390_s_at	pituitary tumor-transforming gene 1	558.1	1839.5	3.3
Purg	1424970_at	purine-rich element binding protein G	572.5	186.8	-3.1
Pvrl3	1448673_at	poliovirus receptor-related 3	383.3	78.3	-4.9
Pzp	1417246_at	pregnancy zone protein	450.8	41.1	-11.0
Rab11fip5	1427405_s_at	RAB11 family interacting protein 5 (class I)	152.3	474.8	3.1
Rab17	1422178_a_at	RAB17, member RAS oncogene family	78.5	321.4	4.1
Rab31	1416165_at	RAB31, member RAS oncogene family	473.9	2080.3	4.4
Rai14	1417400_at	retinoic acid induced 14	926.3	2919.4	3.2
Rarres2	1437902_s_at	retinoic acid receptor responder (tazarotene induced) 2	2562.5	676.6	-3.8
Rasd2	1427344_s_at	RASD family, member 2	346.1	59.7	-5.8
Rasl11b	1423854_a_at	RAS-like, family 11, member B	1086.6	315.7	-3.4
Rbpms	1455936_a_at	RNA binding protein gene with multiple splicing	1001.4	318.1	-3.1
Rbpms2	1424008_a_at	RNA binding protein with multiple splicing 2	1048.1	129.5	-8.1
Rcan2	1421425_a_at	regulator of calcineurin 2	3266.3	173.4	-18.8
Rcn3	1424382_at	reticulocalbin 3, EF-hand calcium binding domain	1203.0	353.9	-3.4
Rdh12	1424256_at	retinol dehydrogenase 12	3112.9	305.2	-10.2
Rdh9	1427963_s_at	retinol dehydrogenase 9	547.5	79.2	-6.9
Reck	1450784_at	reversion-inducing-cysteine-rich protein with kazal motifs	973.6	96.9	-10.1
Reep1	1433509_s_at	receptor accessory protein 1	508.4	101.9	-5.0
Reln	1449465_at	reelin	302.9	85.1	-3.6
Rgl1	1449124_at	ral guanine nucleotide dissociation stimulator,-like 1	325.1	87.6	-3.7
Rgs2	1419248_at	regulator of G-protein signaling 2	2281.3	639.0	-3.6
Rgs4	1416286_at	regulator of G-protein signaling 4	643.7	81.7	-7.9
Rgs5	1417466_at	regulator of G-protein signaling 5	13268.1	2769.3	-4.8
Rhbg	1419134_at	Rhesus blood group-associated B glycoprotein	1040.7	312.9	-3.3
Rhoj	1418892_at	ras homolog gene family, member J	1292.3	296.0	-4.4
Rhpn2	1434628_a_at	rhophilin, Rho GTPase binding protein 2	1029.8	260.6	-4.0
Rin1	1424507_at	Ras and Rab interactor 1	143.5	465.0	3.2
Rnd3	1416700_at	Rho family GTPase 3	398.0	1196.7	3.0
Rod1	1424083_at	ROD1 regulator of differentiation 1 (S. pombe)	335.4	1112.8	3.3
Ror2	1423428_at	receptor tyrosine kinase-like orphan receptor 2	363.4	83.4	-4.4
Rora	1424034_at	RAR-related orphan receptor alpha	1229.7	285.1	-4.3
Rras2	1417398_at	related RAS viral (r-ras) oncogene homolog 2	507.1	2211.9	4.4
Rrm2	1448226_at	ribonucleotide reductase M2	526.6	1962.4	3.7
Rtp4	1418580_at	receptor transporter protein 4	208.8	664.5	3.2
Runx1	1422864_at	runt related transcription factor 1	86.6	1377.3	15.9
Runx2	1424704_at	runt related transcription factor 2	58.4	1095.5	18.7
S100a10	1416762_at	S100 calcium binding protein A10 (calpactin)	2614.0	8840.2	3.4
S100a3	1421856_at	S100 calcium binding protein A3	35.1	620.7	17.7
S100a8	1419394_s_at	S100 calcium binding protein A8 (calgranulin A)	57.0	13047.8	229.0
S100a9	1448756_at	S100 calcium binding protein A9 (calgranulin B)	240.6	17942.3	74.6
S1pr5	1449365_at	sphingosine-1-phosphate receptor 5	558.3	133.0	-4.2
Saa3	1450826_a_at	serum amyloid A 3	117.4	12750.9	108.6
Samhd1	1418131_at	SAM domain and HD domain, 1	449.2	1947.4	4.3
Sat1	1420502_at	spermidine/spermine N1-acetyl transferase 1	2033.4	8609.3	4.2
Scara5	1451204_at	scavenger receptor class A, member 5 (putative)	2088.4	657.5	-3.2
Scd2	1415823_at	stearoyl-Coenzyme A desaturase 2	788.5	3310.5	4.2
Scg2	1450708_at	secretogranin II	328.7	37.1	-8.9
Scgb1a1	1452543_a_at	secretoglobin, family 1A, member 1 (uteroglobin)	4418.7	401.0	-11.0

Scin	1450276_a_at	scinderin	62.0	656.2	10.6
Sdc2	1448545_at	syndecan 2	1316.1	334.1	-3.9
Sdpr	1416778_at	serum deprivation response	3092.7	330.7	-9.4
Sec14l1	1453412_a_at	SEC14-like 1 (S. cerevisiae)	1181.7	382.1	-3.1
Selm	1424394_at	selenoprotein M	1392.8	366.3	-3.8
Sema3e	1419717_at	sema domain, immunoglobulin domain (Ig), short basic domain, secreted, (semaphorin) 3E	944.6	182.8	-5.2
Serpina12	1421092_at	serine (or cysteine) peptidase inhibitor, clade A (alpha-1 antiproteinase, antitrypsin), member 12	6918.5	1099.4	-6.3
Serpina1a	1420553_x_at	serine (or cysteine) peptidase inhibitor, clade A, member 1A	429.1	14.0	-30.6
Serpina1b	1418282_x_at	serine (or cysteine) preptidase inhibitor, clade A, member 1B	667.1	158.8	-4.2
Serpina3k	1423866_at	serine (or cysteine) peptidase inhibitor, clade A, member 3K	1186.0	23.8	-49.8
Serpina3n	1419100_at	serine (or cysteine) peptidase inhibitor, clade A, member 3N	371.6	1996.9	5.4
Serpinb6b	1422804_at	serine (or cysteine) peptidase inhibitor, clade B, member 6b	244.5	787.9	3.2
Serpinb6c	1451594_s_at	serine (or cysteine) peptidase inhibitor, clade B, member 6c	34.8	307.5	8.8
Serpinb7	1449500_at	serine (or cysteine) peptidase inhibitor, clade B, member 7	2546.6	135.0	-18.9
Serpinc1	1417909_at	serine (or cysteine) peptidase inhibitor, clade C (antithrombin), member 1	337.6	64.8	-5.2
Serpine1	1419149_at	serine (or cysteine) peptidase inhibitor, clade E, member 1	82.9	2073.5	25.0
Serpine2	1416666_at	serine (or cysteine) peptidase inhibitor, clade E, member 2	1078.3	3347.6	3.1
Sfrp2	1448201_at	secreted frizzled-related protein 2	2178.8	164.3	-13.3
Sgce	1420688_a_at	sarcoglycan, epsilon	881.9	150.4	-5.9
Sgpl1	1415893_at	sphingosine phosphate lyase 1	806.4	268.6	-3.0
Sh3bgr	1422644_at	SH3-binding domain glutamic acid-rich protein	1374.4	102.7	-13.4
Sh3gl2	1418791_at	SH3-domain GRB2-like 2	627.9	107.4	-5.8
Shcbp1	1416299_at	Shc SH2-domain binding protein 1	218.7	840.0	3.8
Sla	1420819_at	src-like adaptor	118.4	373.5	3.2
Slc15a1	1419343_at	solute carrier family 15 (oligopeptide transporter), member 1	350.8	85.5	-4.1
Slc20a1	1448568_a_at	solute carrier family 20, member 1	536.5	1665.1	3.1
Slc22a23	1453004_at	solute carrier family 22, member 23	2716.4	868.8	-3.1
Slc25a24	1427483_at	solute carrier family 25 (mitochondrial carrier, phosphate carrier), member 24	217.0	841.0	3.9
Slc2a1	1426599_a_at	solute carrier family 2 (facilitated glucose transporter), member 1	1843.9	7547.0	4.1
Slc2a4	1415958_at	solute carrier family 2 (facilitated glucose transporter), member 4	539.4	158.4	-3.4
Slc38a1	1415903_at	solute carrier family 38, member 1	521.2	171.5	-3.0
Slc46a1	1426714_at	solute carrier family 46, member 1	637.8	148.1	-4.3
Slc46a2	1423476_at	solute carrier family 46, member 2	2266.0	352.1	-6.4
Slc6a14	1420503_at	solute carrier family 6 (neurotransmitter transporter), member 14	46.4	728.4	15.7
Slc6a20a	1427221_at	solute carrier family 6 (neurotransmitter transporter), member 20A	250.6	851.5	3.4
Slc7a8	1417929_at	solute carrier family 7 (cationic amino acid transporter, y+ system), member 8	103.8	484.0	4.7
Slfn4	1427102_at	schlafen 4	22.0	561.4	25.5
Smarcd3	1418467_at	SWI/SNF related, matrix associated, actin dependent regulator of chromatin, subfamily d, membe	435.5	77.6	-5.6
Smoc2	1415935_at	SPARC related modular calcium binding 2	1172.8	281.8	-4.2
Smox	1424268_at	spermine oxidase	513.0	1988.3	3.9
Smpd1	1448621_a_at	sphingomyelin phosphodiesterase 1, acid lysosomal	6504.0	1680.0	-3.9
Smpd3	1422779_at	sphingomyelin phosphodiesterase 3, neutral	652.9	154.6	-4.2
Smtn	1452469_a_at	smoothelin	2843.9	353.9	-8.0
Snai2	1418673_at	snail homolog 2 (Drosophila)	150.3	448.8	3.0
Sntg2	1418789_at	syntrophin, gamma 2	667.8	51.3	-13.0
Snx10	1431055_a_at	sorting nexin 10	254.3	800.6	3.1
Socs3	1455899_x_at	suppressor of cytokine signaling 3	720.3	2514.6	3.5
Sorcs2	1419358_at	sortilin-related VPS10 domain containing receptor 2	103.2	343.5	3.3
Sostdc1	1449340_at	sclerostin domain containing 1	90.5	563.1	6.2
Sox6	1427677_a_at	SRY-box containing gene 6	364.8	81.4	-4.5
Sox9	1424950_at	SRY-box containing gene 9	65.3	405.8	6.2
Sparcl1	1416114_at	SPARC-like 1	11730.0	1590.3	-7.4
Spats2l	1425913_a_at	spermatogenesis associated, serine-rich 2-like	947.9	242.0	-3.9
Spc25	1424118_a_at	SPC25, NDC80 kinetochore complex component, homolog (S. cerevisiae)	192.9	577.8	3.0
Speg	1448664_a_at	SPEG complex locus	635.8	72.6	-8.8
Spp1	1449254_at	secreted phosphoprotein 1	1267.2	7091.8	5.6
Sprr1b	1422672_at	small proline-rich protein 1B	244.1	6903.4	28.3
Sprr2f	1449833_at	small proline-rich protein 2F	180.3	10147.4	56.3
Sprr2h	1422240_s_at	small proline-rich protein 2H	115.7	9786.3	84.6
Sprr2k	1422425_at	small proline-rich protein 2K	327.4	1475.0	4.5
Srgn	1417426_at	serglycin	1007.6	8887.1	8.8
Srpx	1451939_a_at	sushi-repeat-containing protein	463.7	103.4	-4.5
Sspn	1417644_at	sarcospan	727.7	188.6	-3.9
St3gal4	1425668_a_at	ST3 beta-galactoside alpha-2,3-sialyltransferase 4	53.0	852.7	16.1
Star	1418728_at	steroidogenic acute regulatory protein	451.4	108.9	-4.1
Stard5	1422821_s_at	StAR-related lipid transfer (START) domain containing 5	1900.4	591.7	-3.2
Stfa3	1419709_at	stefin A3	104.2	3573.4	34.3
Stk39	1419551_s_at	serine/threonine kinase 39, STE20/SPS1 homolog (yeast)	460.7	1532.0	3.3
Stom	1438910_a_at	stomatin	947.1	4707.9	5.0
Stra6	1422723_at	stimulated by retinoic acid gene 6	43.8	383.3	8.8
Sult1a1	1427345_a_at	sulfotransferase family 1A, phenol-preferring, member 1	434.9	138.6	-3.1
Sult1b1	1418940_at	sulfotransferase family 1B, member 1	617.9	156.8	-3.9

Sult1c2	1449409_at	sulfotransferase family, cytosolic, 1C, member 2	4361.1	1124.6	-3.9
Sult1d1	1448973_at	sulfotransferase family 1D, member 1	1812.0	377.2	-4.8
Suox	1451339_at	sulfite oxidase	1135.7	255.9	-4.4
Susd2	1426511_at	sushi domain containing 2	638.3	111.5	-5.7
Svil	1460694_s_at	supervillin	3388.9	1100.8	-3.1
Syncrip	1422768_at	synaptotagmin binding, cytoplasmic RNA interacting protein	144.7	500.4	3.5
Synpo2	1450828_at	synaptopodin 2	352.2	111.1	-3.2
Sytl2	1421594_a_at	synaptotagmin-like 2	1095.6	237.4	-4.6
Tac1	1416783_at	tachykinin 1	1135.4	98.0	-11.6
Tacc3	1417450_a_at	transforming, acidic coiled-coil containing protein 3	109.3	490.0	4.5
Tagln	1423505_at	transgelin	24173.5	2523.7	-9.6
Tceal1	1424634_at	transcription elongation factor A (SII)-like 1	343.2	94.6	-3.6
Tcf21	1417447_at	transcription factor 21	899.9	135.5	-6.6
Tcf23	1421078_at	transcription factor 23	1284.9	386.1	-3.3
Tex15	1420719_at	testis expressed gene 15	125.9	517.0	4.1
Tgfb1i1	1418136_at	transforming growth factor beta 1 induced transcript 1	1690.6	190.3	-8.9
Tgif1	1422286_a_at	TGFB-induced factor homeobox 1	270.7	1055.5	3.9
Tgm2	1433428_x_at	transglutaminase 2, C polypeptide	5851.7	1733.3	-3.4
Tgm3	1421355_at	transglutaminase 3, E polypeptide	1745.4	267.4	-6.5
Thbs1	1460302_at	thrombospondin 1	244.2	1845.4	7.6
Thbs3	1416623_at	thrombospondin 3	304.6	39.1	-7.8
Thoc4	1417724_at	THO complex 4	326.6	995.3	3.0
Thop1	1448907_at	thimet oligopeptidase 1	2296.2	369.8	-6.2
Thrsp	1424737_at	thyroid hormone responsive SPOT14 homolog (Rattus)	342.0	25.1	-13.6
Timp1	1460227_at	tissue inhibitor of metalloproteinase 1	122.2	2287.1	18.7
Timp2	1433662_s_at	tissue inhibitor of metalloproteinase 2	4173.2	1088.4	-3.8
Tln1	1448402_at	talin 1	1406.8	461.3	-3.1
Tm4sf1	1450958_at	transmembrane 4 superfamily member 1	852.5	5372.4	6.3
Tm4sf5	1424445_at	transmembrane 4 superfamily member 5	480.9	108.4	-4.4
Tmcc3	1451794_at	transmembrane and coiled coil domains 3	1015.8	264.7	-3.8
Tmem47	1420514_at	transmembrane protein 47	1498.3	289.1	-5.2
Tmigd1	1419498_at	transmembrane and immunoglobulin domain containing 1	267.1	2705.1	10.1
Tnfaip2	1438855_x_at	tumor necrosis factor, alpha-induced protein 2	637.7	3160.3	5.0
Tnfrsf19	1448147_at	tumor necrosis factor receptor superfamily, member 19	1020.1	215.4	-4.7
Tnfrsf22	1426095_a_at	tumor necrosis factor receptor superfamily, member 22	95.8	394.2	4.1
Tnnc2	1417464_at	troponin C2, fast	25.5	861.4	33.8
Tnnt2	1418726_a_at	troponin T2, cardiac	159.0	779.7	4.9
Tns1	1419283_s_at	tensin 1	4418.9	298.4	-14.8
Tnxb	1450798_at	tenascin XB	742.7	74.1	-10.0
Top2a	1454694_a_at	topoisomerase (DNA) II alpha	537.3	1818.7	3.4
Tpbg	1423310_at	trophoblast glycoprotein	135.7	589.6	4.3
Tpm1	1423721_at	tropomyosin 1, alpha	15625.4	4467.7	-3.5
Tpp2	1430575_a_at	tripeptidyl peptidase II	322.1	70.6	-4.6
Tppp3	1416713_at	tubulin polymerization-promoting protein family member 3	2064.0	99.2	-20.8
Tpx2	1428104_at	TPX2, microtubule-associated protein homolog (Xenopus laevis)	107.2	445.4	4.2
Trib2	1426640_s_at	tribbles homolog 2 (Drosophila)	196.8	759.5	3.9
Trip13	1429295_s_at	thyroid hormone receptor interactor 13	261.9	918.1	3.5
Tspan13	1418643_at	tetraspanin 13	3639.3	1070.6	-3.4
Tspan15	1424653_at	tetraspanin 15	664.9	204.4	-3.3
Tspan2	1424567_at	tetraspanin 2	1422.0	183.5	-7.8
Tspan8	1420019_at	tetraspanin 8	569.6	162.5	-3.5
Tspyl4	1424029_at	TSPY-like 4	1032.2	124.9	-8.3
Tst	1448609_at	thiosulfate sulfurtransferase, mitochondrial	2439.6	485.8	-5.0
Ttc28	1419584_at	tetratricopeptide repeat domain 28	548.0	116.4	-4.7
Ttc36	1418696_at	tetratricopeptide repeat domain 36	91.4	317.9	3.5
Ttpa	1427284_a_at	tocopherol (alpha) transfer protein	539.4	105.3	-5.1
Ttr	1454608_x_at	transthyretin	1929.3	222.8	-8.7
Tug1	1456398_at	taurine upregulated gene 1	321.4	90.7	-3.5
Tyrobp	1450792_at	TYRO protein tyrosine kinase binding protein	420.9	2361.6	5.6
Ube2c	1452954_at	ubiquitin-conjugating enzyme E2C	405.3	1756.6	4.3
Ubl7	1451465_at	ubiquitin-like 7 (bone marrow stromal cell-derived)	319.2	89.4	-3.6
Ugt2b34	1427961_s_at	UDP glucuronosyltransferase 2 family, polypeptide B34	2182.4	446.9	-4.9
Uox	1422604_at	urate oxidase	114.4	406.0	3.5
Upk3b	1454881_s_at	uroplakin 3B	528.0	56.6	-9.3
Upp1	1448562_at	uridine phosphorylase 1	260.1	3358.3	12.9
Usp2	1417168_a_at	ubiquitin specific peptidase 2	552.7	61.7	-9.0
Vamp5	1430522_a_at	vesicle-associated membrane protein 5	220.9	1586.1	7.2
Vasn	1455812_x_at	vasorin	2590.3	845.4	-3.1
Vcam1	1448162_at	vascular cell adhesion molecule 1	120.5	431.3	3.6
Vegfa	1420909_at	vascular endothelial growth factor A	250.6	1064.6	4.2
Vegfb	1451803_a_at	vascular endothelial growth factor B	482.9	153.4	-3.1
Vil1	1448837_at	villin 1	351.2	48.8	-7.2

Vip	1428664_at	vasoactive intestinal polypeptide	913.2	216.3	-4.2
Vnn1	1418486_at	vanin 1	1264.5	7278.7	5.8
Vsig2	1422634_a_at	V-set and immunoglobulin domain containing 2	1384.3	386.1	-3.6
Vsnl1	1420955_at	visinin-like 1	194.0	670.1	3.5
Wfdc1	1431335_a_at	WAP four-disulfide core domain 1	1023.3	225.3	-4.5
Wfdc2	1424351_at	WAP four-disulfide core domain 2	543.1	6500.7	12.0
Wnk2	1453355_at	WNK lysine deficient protein kinase 2	387.1	81.6	-4.7
Wnk4	1427196_at	WNK lysine deficient protein kinase 4	595.0	149.8	-4.0
Wnt5a	1436791_at	wingless-related MMTV integration site 5A	135.7	534.4	3.9
Zbtb20	1438443_at	zinc finger and BTB domain containing 20	790.6	184.8	-4.3
Zeb1	1418926_at	zinc finger E-box binding homeobox 1	1205.9	346.5	-3.5
Zfp52	1426471_at	zinc finger protein 52	344.6	1730.5	5.0
Zfp574	1434128_a_at	zinc finger protein 574	923.4	308.0	-3.0
Zfp7	1451323_at	zinc finger protein 7	118.5	377.0	3.2
Zfp772	1460381_at	zinc finger protein 772	806.6	262.7	-3.1
Zfpm2	1449314_at	zinc finger protein, multitype 2	363.0	42.5	-8.5
Zranb1	1415712_at	zinc finger, RAN-binding domain containing 1	978.0	317.6	-3.1
Zwilch	1416757_at	Zwilch, kinetochore associated, homolog (Drosophila)	99.7	350.8	3.5
