# Supplementary material for: Induction of Metastatic Gastric Cancer by Peroxisome Proliferator-Activated Receptorδ Activation
Source: PPAR Res. 2010 Dec 27;2010:571783. doi: 10.1155/2010/571783 (PMC3026990; doi:10.1155/2010/571783)
Supplement: Supplementary file 3 [file 571783.f3.pdf]

TABLE S3. Differentially expressed genes in the stomach after GW501516 treatment compared to untreated stomach

Gene Symbol	Probe set	Gene Name	Stomach	+GW510516	Fold Change
Adipoq	1422651_at	adiponectin, C1Q and collagen domain containing	337.6	923.8	3.0
Agr2	1419268_at	anterior gradient 2 (Xenopus laevis)	5897.7	923.4	-6.4
Alb	1425260_at	albumin	6338.0	953.9	-6.6
Angptl4	1417130_s_at	angiopoietin-like 4	272.6	766.5	3.0
Apoa2	1417950_a_at	apolipoprotein A-II	630.5	136.3	-4.6
Apoh	1416677_at	apolipoprotein H	540.8	126.0	-4.3
Avil	1419148_at	advillin	1430.4	433.5	-3.3
Cela1	1423693_at	chymotrypsin-like elastase family, member 1	4573.7	351.4	-13.0
Cfd	1417867_at	complement factor D (adipsin)	310.2	875.6	3.0
Chgb	1415885_at	chromogranin B	1117.8	165.9	-6.7
Chi3l4	1425450_at	chitinase 3-like 4	476.3	1200.6	3.0
Clca3	1416306_at	chloride channel calcium activated 3	2163.8	39.4	-54.9
Cldn18	1449428_at	claudin 18	3103.4	963.7	-3.2
Cldn2	1417231_at	claudin 2	572.0	46.2	-12.4
Cpn1	1417745_at	carboxypeptidase N, polypeptide 1	313.3	55.3	-5.7
Cyp2b10	1425645_s_at	cytochrome P450, family 2, subfamily b, polypeptide 10	100.6	344.6	3.4
Cyp3a11	1416809_at	cytochrome P450, family 3, subfamily a, polypeptide 11	497.9	49.2	-10.1
Dmbt1	1418287_a_at	deleted in malignant brain tumors 1	2654.7	288.4	-9.2
Fabp2	1418438_at	fatty acid binding protein 2, intestinal	1999.0	209.4	-9.5
Fcgbp	1426872_at	Fc fragment of IgG binding protein	652.8	128.8	-5.1
Foxa2	1422833_at	forkhead box A2	359.8	116.7	-3.1
Gast	1422915_at	gastrin	6048.0	56.8	-106.5
Gc	1426547_at	group specific component	531.6	71.4	-7.4
Gcnt3	1424901_at	glucosaminyl (N-acetyl) transferase 3, mucin type	791.1	211.1	-3.7
Glycam1	1424825_a_at	glycosylation dependent cell adhesion molecule 1	1274.4	29.6	-43.1
Gstm3	1427474_s_at	glutathione S-transferase, mu 3	1125.7	363.9	-3.1
Hpx	1423944_at	hemopexin	439.7	64.1	-6.9
Kcnk1	1448690_at	potassium channel, subfamily K, member 1	750.3	242.9	-3.1
Krt18	1448169_at	keratin 18	3170.6	1227.7	-3.0
Krt19	1417156_at	keratin 19	11777.1	3738.6	-3.2
Krt20	1426284_at	keratin 20	1764.8	238.0	-7.4
Krt7	1423952_a_at	keratin 7	1225.9	375.1	-3.3
Krt8	1423691_x_at	keratin 8	10878.5	2818.7	-3.9
Lgals4	1451336_at	lectin, galactose binding, soluble 4	2691.5	874.9	-3.1
Mal	1417275_at	myelin and lymphocyte protein, T-cell differentiation protein	4462.8	1375.1	-3.2
Muc5ac	1430899_at	mucin 5, subtypes A and C, tracheobronchial/gastric	11100.2	1380.6	-8.0
Pah	1454638_a_at	phenylalanine hydroxylase	358.9	34.5	-10.4
Pglyrp1	1449184_at	peptidoglycan recognition protein 1	403.0	127.5	-3.2
Pigr	1455490_at	polymeric immunoglobulin receptor	324.0	53.0	-6.1
Pls1	1460406_at	plastin 1 (I-isoform)	1419.8	453.6	-3.1
Prom1	1419700_a_at	prominin 1	1316.6	404.6	-3.3
Pzp	1417246_at	pregnancy zone protein	450.8	54.3	-8.3
Serpina1a	1420553_x_at	serine (or cysteine) peptidase inhibitor, clade A, member 1A	429.1	32.6	-13.2
Serpina1b	1418282_x_at	serine (or cysteine) preptidase inhibitor, clade A, member 1B	667.1	111.1	-6.0
Serpina3k	1423866_at	serine (or cysteine) peptidase inhibitor, clade A, member 3K	1186.0	143.1	-8.3
Serpinc1	1417909_at	serine (or cysteine) peptidase inhibitor, clade C (antithrombin), member 1	337.6	104.0	-3.2
Slc39a4	1451139_at	solute carrier family 39 (zinc transporter), member 4	327.8	110.1	-3.0
Spink4	1427119_at	serine peptidase inhibitor, Kazal type 4	654.4	133.4	-4.9
Spp1	1449254_at	secreted phosphoprotein 1	1267.2	70.8	-17.9
Sult1d1	1448973_at	sulfotransferase family 1D, member 1	1812.0	623.1	-3.0
Tff2	1422448_at	trefoil factor 2 (spasmolytic protein 1)	15520.2	2635.3	-5.9
Ttr	1455913_x_at	transthyretin	1357.6	410.6	-3.3
Vil1	1448837_at	villin 1	351.2	39.4	-8.9
