# Supplementary material for: Induction of Metastatic Gastric Cancer by Peroxisome Proliferator-Activated Receptorδ Activation
Source: PPAR Res. 2010 Dec 27;2010:571783. doi: 10.1155/2010/571783 (PMC3026990; doi:10.1155/2010/571783)
Supplement: Supplementary file 4 [file 571783.f4.pdf]

TABLE S4. Differentially expressed genes in the stomach after treatment with DMBA compared to untreated stomach.

Gene Symbol	Probe set	Gene Name	Stomach	+DMBA	Fold Change
Acpp	1419832_s_at	acid phosphatase, prostate	279.4	84.2	-3.3
Acss1	1416617_at	acyl-CoA synthetase short-chain family member 1	741.5	238.1	-3.1
Adamdec1	1419476_at	ADAM-like, decysin 1	358.8	59.5	-6.0
Adipoq	1422651_at	adiponectin, C1Q and collagen domain containing	337.6	41.9	-8.1
Agr2	1419268_at	anterior gradient 2 (Xenopus laevis)	5897.7	90.7	-65.0
Aif1l	1424263_at	allograft inflammatory factor 1-like	1759.1	273.5	-6.4
Akr1c12	1422000_at	aldo-keto reductase family 1, member C12	1185.1	93.0	-12.7
Akr1c13	1418672_at	aldo-keto reductase family 1, member C13	825.5	116.9	-7.1
Akr1c14	1418979_at	aldo-keto reductase family 1, member C14	673.1	96.5	-7.0
Alb	1425260_at	albumin	6338.0	436.8	-14.5
Anxa10	1449426_a_at	annexin A10	13369.8	448.1	-29.8
Apoa1	1419232_a_at	apolipoprotein A-I	1640.1	312.5	-5.2
Apoa2	1417950_a_at	apolipoprotein A-II	630.5	104.9	-6.0
Apoh	1416677_at	apolipoprotein H	540.8	84.5	-6.4
Areg	1421134_at	amphiregulin	291.0	66.2	-4.4
Atf3	1449363_at	activating transcription factor 3	351.1	97.4	-3.6
Atp2a3	1421129_a_at	ATPase, Ca++ transporting, ubiquitous	1366.8	438.2	-3.1
Atp4a	1421286_a_at	ATPase, H+/K+ exchanging, gastric, alpha polypeptide	2423.5	365.8	-6.6
Atp4b	1448911_at	ATPase, H+/K+ exchanging, beta polypeptide	3882.2	536.2	-7.2
Avil	1419148_at	advillin	1430.4	120.2	-11.9
Bace2	1438645_x_at	beta-site APP-cleaving enzyme 2	1662.4	429.2	-3.9
Btc	1435541_at	betacellulin, epidermal growth factor family member	592.9	187.2	-3.2
Car3	1449434_at	carbonic anhydrase 3	1289.8	135.2	-9.5
Casp6	1415995_at	caspase 6	1277.9	426.8	-3.0
Ccl8	1419684_at	chemokine (C-C motif) ligand 8	101.9	475.6	4.7
Ceacam1	1425538_x_at	carcinoembryonic antigen-related cell adhesion molecule 1	235.8	67.4	-3.5
Cela1	1423693_at	chymotrypsin-like elastase family, member 1	4573.7	255.6	-17.9
Cfd	1417867_at	complement factor D (adipsin)	310.2	78.0	-4.0
Chga	1418149_at	chromogranin A	361.5	74.0	-4.9
Chgb	1415885_at	chromogranin B	1117.8	110.5	-10.1
Cideb	1418976_s_at	cell death-inducing DNA fragmentation factor, alpha subunit-like effector B	331.2	94.6	-3.5
Clca3	1416306_at	chloride channel calcium activated 3	2163.8	38.4	-56.3
Cldn18	1449428_at	claudin 18	3103.4	137.0	-22.6
Cldn2	1417231_at	claudin 2	572.0	37.6	-15.2
Cldn7	1448393_at	claudin 7	177.9	38.7	-4.6
Cpn1	1417745_at	carboxypeptidase N, polypeptide 1	313.3	45.3	-6.9
Ctse	1418989_at	cathepsin E	15476.1	653.1	-23.7
Cxcl13	1417851_at	chemokine (C-X-C motif) ligand 13	239.0	46.9	-5.1
Cxcl17	1451610_at	chemokine (C-X-C motif) ligand 17	562.3	106.1	-5.3
Cyp2c65	1429994_s_at	cytochrome P450, family 2, subfamily c, polypeptide 65	1523.7	116.0	-13.1
Cyp2e1	1415994_at	cytochrome P450, family 2, subfamily e, polypeptide 1	646.8	65.0	-10.0
Cyp3a11	1416809_at	cytochrome P450, family 3, subfamily a, polypeptide 11	497.9	153.9	-3.2
Defb3	1421806_at	defensin beta 3	104.2	443.9	4.3
Degs2	1424549_at	degenerative spermatocyte homolog 2 (Drosophila), lipid desaturase	2460.9	515.5	-4.8
Dmbt1	1418287_a_at	deleted in malignant brain tumors 1	2654.7	57.6	-46.1
Dsc3	1427637_a_at	desmocollin 3	199.9	637.2	3.2
Dsg2	1449740_s_at	desmoglein 2	1715.4	343.0	-5.0
Eepd1	1417877_at	endonuclease/exonuclease/phosphatase family domain containing 1	458.3	123.2	-3.7
Elf3	1416916_at	E74-like factor 3	779.1	191.1	-4.1
Fabp1	1448764_a_at	fatty acid binding protein 1, liver	846.0	176.0	-4.8
Fabp2	1418438_at	fatty acid binding protein 2, intestinal	1999.0	434.1	-4.6
Fam3b	1425122_at	family with sequence similarity 3, member B	727.7	101.5	-7.2
Fetub	1449555_a_at	fetuin beta	5683.2	919.4	-6.2
Fos	1423100_at	FBJ osteosarcoma oncogene	1503.0	180.0	-8.4
Foxa2	1422833_at	forkhead box A2	359.8	41.8	-8.6
Foxa3	1431900_a_at	forkhead box A3	342.2	27.1	-12.6
Foxq1	1422735_at	forkhead box Q1	759.2	227.6	-3.3
Galnt4	1455915_at	UDP-N-acetyl-alpha-D-galactosamine:polypeptide N-acetylgalactosaminyltransferase 4	1340.9	418.9	-3.2
Gast	1422915_at	gastrin	6048.0	53.5	-113.1
Gata6	1425464_at	GATA binding protein 6	334.2	96.7	-3.5
Gba2	1434271_at	glucosidase beta 2	1074.8	361.0	-3.0
Gc	1426547_at	group specific component	531.6	36.9	-14.4
Gcnt3	1424901_at	glucosaminyl (N-acetyl) transferase 3, mucin type	791.1	42.3	-18.7
Ghrl	1448980_at	ghrelin	1425.0	80.3	-17.7
Gif	1419020_at	gastric intrinsic factor	3624.1	388.8	-9.3
Gipc2	1417178_at	GIPC PDZ domain containing family, member 2	1256.4	173.8	-7.2
Gja1	1415801_at	gap junction protein, alpha 1	865.6	4146.8	4.8
Gjb1	1448766_at	gap junction protein, beta 1	557.6	165.0	-3.4
Gkn1	1423404_at	gastrokine 1	30348.0	6969.0	-4.4
Gkn2	1453132_a_at	gastrokine 2	35766.4	10991.3	-3.3

Glycam1	1424825_a_at	glycosylation dependent cell adhesion molecule 1	1274.4	27.0	-47.1
Golm1	1415698_at	golgi membrane protein 1	2712.5	308.1	-8.8
Gpa33	1419330_a_at	glycoprotein A33 (transmembrane)	175.3	53.4	-3.3
Gstm3	1427474_s_at	glutathione S-transferase, mu 3	1125.7	378.5	-3.0
Hnf4a	1427001_s_at	hepatic nuclear factor 4, alpha	624.0	104.0	-6.0
Hpx	1423944_at	hemopexin	439.7	54.4	-8.1
Hsd17b11	1421011_at	hydroxysteroid (17-beta) dehydrogenase 11	857.4	230.5	-3.7
Iqgap2	1459894_at	IQ motif containing GTPase activating protein 2	361.3	81.2	-4.4
Isl1	1450723_at	ISL1 transcription factor, LIM/homeodomain	157.9	42.6	-3.7
Iyd	1451547_at	iodotyrosine deiodinase	480.6	43.8	-11.0
Kcnk1	1448690_at	potassium channel, subfamily K, member 1	750.3	222.3	-3.4
Kcnn4	1435945_a_at	potassium intermediate/small conductance calcium-activated channel, subfamily N, member 4	629.8	154.9	-4.1
Kctd4	1420537_at	potassium channel tetramerisation domain containing 4	615.1	64.8	-9.5
Krt16	1448932_at	keratin 16	86.8	1412.1	16.3
Krt18	1448169_at	keratin 18	3170.6	248.1	-12.8
Krt19	1417156_at	keratin 19	11777.1	858.9	-13.7
Krt20	1426284_at	keratin 20	1764.8	74.9	-23.6
Krt6a	1422784_at	keratin 6A	241.2	1936.9	8.0
Krt6b	1422588_at	keratin 6B	2756.1	10196.9	3.7
Krt7	1423952_a_at	keratin 7	1225.9	268.0	-4.6
Krt8	1420647_a_at	keratin 8	16262.0	670.3	-24.3
Lce1a1	1420677_x_at	late cornified envelope 1A1	15780.9	3119.0	-5.1
Lce1a2	1420350_at	late cornified envelope 1A2	18579.5	1843.3	-10.1
Lce1b	1419409_at	late cornified envelope 1B	8715.4	715.3	-12.2
Lce1d	1420332_x_at	late cornified envelope 1D	16703.6	1936.1	-8.6
Lce1f	1420550_at	late cornified envelope 1F	19029.5	4469.3	-4.3
Lce1g	1421316_at	late cornified envelope 1G	312.3	47.3	-6.6
Lce1h	1449959_x_at	late cornified envelope 1H	20812.5	1768.1	-11.8
Lce1l	1418855_at	late cornified envelope 1L	1186.9	37.9	-31.3
Lce3a	1456001_at	late cornified envelope 3A	10378.6	2352.4	-4.4
Lgals2	1417078_at	lectin, galactose-binding, soluble 2	2675.4	306.8	-8.7
Lgals4	1451336_at	lectin, galactose binding, soluble 4	2691.5	192.2	-14.0
Lgals9	1421217_a_at	lectin, galactose binding, soluble 9	2077.3	663.2	-3.1
Lmo4	1420981_a_at	LIM domain only 4	1147.4	383.6	-3.0
Lnx1	1450251_a_at	ligand of numb-protein X 1	406.0	71.2	-5.7
Lrrc26	1451498_at	leucine rich repeat containing 26	414.8	46.8	-8.9
Ltf	1450009_at	lactotransferrin	163.3	23.6	-6.9
Mal	1417275_at	myelin and lymphocyte protein, T-cell differentiation protein	4462.8	162.4	-27.5
Me2	1426572_at	malic enzyme 2, NAD(+)-dependent, mitochondrial	1292.3	387.8	-3.3
Mlph	1449896_at	melanophilin	472.5	28.2	-16.7
Muc1	1449199_at	mucin 1, transmembrane	884.7	75.8	-11.7
Muc5ac	1430899_at	mucin 5, subtypes A and C, tracheobronchial/gastric	11100.2	683.3	-16.2
Myh14	1428835_at	myosin, heavy polypeptide 14	557.4	178.3	-3.1
Myo5c	1424933_at	myosin VC	606.9	119.9	-5.1
Nt5c3	1451050_at	5'-nucleotidase, cytosolic III	3347.1	1084.9	-3.1
Oasl1	1424339_at	2'-5' oligoadenylate synthetase-like 1	604.9	96.6	-6.3
Pah	1454638_a_at	phenylalanine hydroxylase	358.9	29.5	-12.2
Papss2	1434510_at	3'-phosphoadenosine 5'-phosphosulfate synthase 2	2583.6	562.8	-4.6
Pcp4	1460214_at	Purkinje cell protein 4	313.1	69.9	-4.5
Pga5	1421113_at	pepsinogen 5, group I	733.1	101.8	-7.2
Pgc	1415786_at	progastricsin (pepsinogen C)	1236.8	301.0	-4.1
Pglyrp1	1449184_at	peptidoglycan recognition protein 1	403.0	47.7	-8.4
Pigr	1450060_at	polymeric immunoglobulin receptor	278.2	21.1	-13.2
Pla2g10	1451502_at	phospholipase A2, group X	1314.9	78.6	-16.7
Pla2g12b	1419614_at	phospholipase A2, group XIIB	377.1	105.6	-3.6
Plac8	1451335_at	placenta-specific 8	2765.5	664.1	-4.2
Pllp	1448945_at	plasma membrane proteolipid	1075.1	118.1	-9.1
Pls1	1460406_at	plastin 1 (I-isoform)	1419.8	93.3	-15.2
Prom1	1419700_a_at	prominin 1	1316.6	158.9	-8.3
Psca	1451258_at	prostate stem cell antigen	8876.8	982.3	-9.0
Psors1c2	1420467_at	psoriasis susceptibility 1 candidate 2 (human)	492.6	136.9	-3.6
Pzp	1417246_at	pregnancy zone protein	450.8	34.9	-12.9
Rep15	1420328_at	RAB15 effector protein	1733.7	152.2	-11.4
Rhpn2	1434628_a_at	rhophilin, Rho GTPase binding protein 2	1029.8	254.0	-4.1
Rnasel	1426604_at	ribonuclease L (2', 5'-oligoisoadenylate synthetase-dependent)	558.2	146.3	-3.8
Rnd3	1416701_at	Rho family GTPase 3	263.4	784.5	3.0
Rnf128	1449036_at	ring finger protein 128	1717.3	256.1	-6.7
S100a8	1419394_s_at	S100 calcium binding protein A8 (calgranulin A)	57.0	543.2	9.5
S100a9	1448756_at	S100 calcium binding protein A9 (calgranulin B)	240.6	867.6	3.6
Scamp5	1451224_at	secretory carrier membrane protein 5	302.7	80.1	-3.8
Scd2	1415824_at	stearoyl-Coenzyme A desaturase 2	151.4	451.7	3.0
Sdcbp2	1424090_at	syndecan binding protein (syntenin) 2	3418.6	615.0	-5.6

Sectm1b	1419478_at	secreted and transmembrane 1B	327.6	61.4	-5.3
Serpina1a	1420553_x_at	serine (or cysteine) peptidase inhibitor, clade A, member 1A	429.1	19.9	-21.5
Serpina1b	1418282_x_at	serine (or cysteine) preptidase inhibitor, clade A, member 1B	667.1	79.6	-8.4
Serpina3k	1423866_at	serine (or cysteine) peptidase inhibitor, clade A, member 3K	1186.0	24.1	-49.1
Serpina3n	1419100_at	serine (or cysteine) peptidase inhibitor, clade A, member 3N	371.6	1564.5	4.2
Serpinc1	1417909_at	serine (or cysteine) peptidase inhibitor, clade C (antithrombin), member 1	337.6	68.1	-5.0
Slc39a4	1451139_at	solute carrier family 39 (zinc transporter), member 4	327.8	57.5	-5.7
Slc44a4	1416596_at	solute carrier family 44, member 4	710.2	151.4	-4.7
Slc45a3	1426664_x_at	solute carrier family 45, member 3	405.7	111.1	-3.7
Slc45a3	1426663_s_at	solute carrier family 45, member 3	540.3	103.0	-5.2
Socs3	1456212_x_at	suppressor of cytokine signaling 3	369.7	122.1	-3.0
Spink4	1427119_at	serine peptidase inhibitor, Kazal type 4	654.4	41.3	-15.9
Spp1	1449254_at	secreted phosphoprotein 1	1267.2	95.1	-13.3
Sprr1b	1422672_at	small proline-rich protein 1B	244.1	764.2	3.1
Sprr2f	1449833_at	small proline-rich protein 2F	180.3	567.1	3.1
Sprr2h	1422240_s_at	small proline-rich protein 2H	115.7	453.2	3.9
Sst	1417954_at	somatostatin	322.4	65.8	-4.9
Star	1418728_at	steroidogenic acute regulatory protein	451.4	91.8	-4.9
Stfa3	1419709_at	stefin A3	104.2	835.4	8.0
Sult1b1	1418940_at	sulfotransferase family 1B, member 1	617.9	41.4	-14.9
Sult1c2	1449409_at	sulfotransferase family, cytosolic, 1C, member 2	4361.1	155.2	-28.1
Sult1d1	1448973_at	sulfotransferase family 1D, member 1	1812.0	73.7	-24.6
Syt8	1450800_at	synaptotagmin VIII	439.0	147.3	-3.0
Sytl2	1421594_a_at	synaptotagmin-like 2	1095.6	276.9	-4.0
Tcfcp2l1	1418091_at	transcription factor CP2-like 1	552.4	182.1	-3.0
Tff1	1448156_at	trefoil factor 1	28132.6	6943.3	-4.1
Tff2	1422448_at	trefoil factor 2 (spasmolytic protein 1)	15520.2	244.7	-63.4
Tm4sf5	1424445_at	transmembrane 4 superfamily member 5	480.9	116.0	-4.1
Tmprss2	1419154_at	transmembrane protease, serine 2	768.9	69.9	-11.0
Trp63	1451876_a_at	transformation related protein 63	115.2	366.4	3.2
Tspan1	1417957_a_at	tetraspanin 1	1292.5	79.7	-16.2
Tspan8	1424649_a_at	tetraspanin 8	4066.7	164.9	-24.7
Ttr	1455913_x_at	transthyretin	1357.6	71.5	-19.0
U46068	1439423_x_at	cDNA sequence U46068	346.2	79.2	-4.4
Ugt2b34	1427961_s_at	UDP glucuronosyltransferase 2 family, polypeptide B34	2182.4	388.2	-5.6
Vil1	1448837_at	villin 1	351.2	26.2	-13.4
Vsig2	1422634_a_at	V-set and immunoglobulin domain containing 2	1384.3	79.0	-17.5
Wfdc2	1424351_at	WAP four-disulfide core domain 2	543.1	97.3	-5.6
